# *Pnky* lncRNA secondary structure maps identify functional regions that control neurogenesis in neural stem cells

**DOI:** 10.1016/j.celrep.2026.117451

**Published:** 2026-05-28

**Authors:** Parna Saha, Shivali Patel, Lucille H. Tsao, Rafael CA. Tavares, Hyeonseok Choi, Rebecca E. Andersen, Anna Marie Pyle, Daniel A. Lim

**Affiliations:** 1 Department of Neurological Surgery, University of California, San Francisco, San Francisco, CA 94143, USA; 2 Eli and Edythe Broad Center of Regeneration Medicine and Stem Cell Research, University of California, San Francisco, San Francisco, CA 94143, USA; 3 San Francisco Veterans Affairs Medical Center, San Francisco, CA 94121, USA; 4 Department of Molecular Biophysics and Biochemistry, Yale University, New Haven, CT 06511, USA; 5 Department of Chemistry, Yale University, New Haven, CT 06511, USA; 6 Cancer Research UK Cambridge Institute, University of Cambridge, Cambridge, UK; 7 Department of Molecular and Cell Biology Undergraduate Program, University of California, Berkeley, Berkeley, CA 94720, USA; 8 Division of Genetics and Genomics, Harvard Medical School, Boston Children’s Hospital, Boston, MA 02115, USA; 9 Department of Molecular, Cellular, and Developmental Biology, Yale University, New Haven, CT 06511, USA; 10 Howard Hughes Medical Institute, Chevy Chase, MD 20815, USA; 11 These authors contributed equally; 12 Lead contact

## Abstract

Long noncoding RNA (lncRNA) *Pnky* is a *trans-*acting regulator of neural stem cell (NSC) differentiation, but the molecular mechanisms by which *Pnky* regulates neurogenesis are unknown. A fundamental step toward mechanistic understanding is to determine whether lncRNA structure underlies biological function. Using chemical probing and high-throughput analysis, we determined the secondary structure of *Pnky* folded *in vitro* and *in cellulo*. *In vitro*-transcribed *Pnky* RNA adopts a compact, highly structured conformation with evidence of tertiary interactions. *In cellulo, Pnky* secondary structure is similar to the *in vitro* conformation. We used locked nucleic acid (LNA) oligonucleotides to interrogate the entire *Pnky* transcript for function in NSCs and identified regions that when targeted increase neurogenesis—phenocopying *Pnky* knockdown—without decreasing transcript abundance. Our findings implicate specific structured regions of *Pnky* in the regulation of neurogenesis and illustrate how structural maps combined with phenotypic data can advance our understanding of lncRNA function.

## In brief

Saha et al. map the secondary structure and tertiary interactions of the lncRNA *Pnky* and identify structured regions that regulate neurogenesis from neural stem cells.

## Graphical Abstract

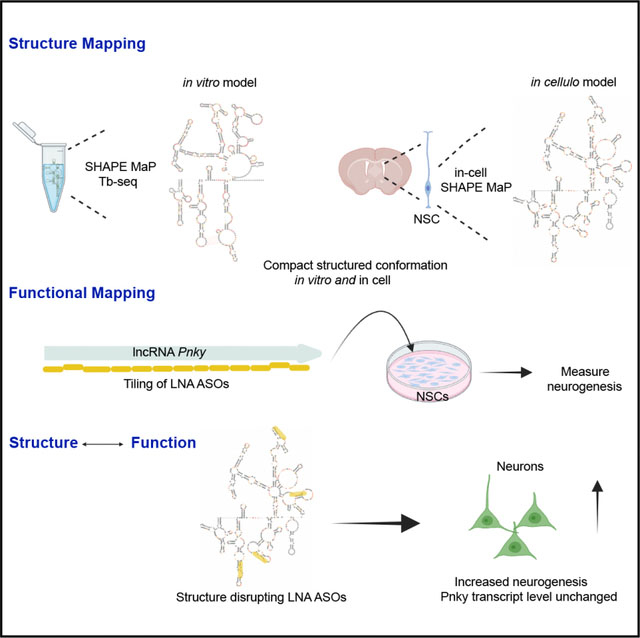

## INTRODUCTION

Neurogenesis—the birth of new neurons from neural stem cells (NSCs)—is a highly orchestrated process that involves the function of numerous genes, some of which “restrain” neuronal production, ensuring that proper numbers of neurons populate the brain across the continuum of development.^1–3^ In recent years, a growing number of long noncoding RNAs (lncRNAs)—transcripts longer than 200 nucleotides (nts) that do not code for protein—have been identified as regulators of neurogenesis.^4,5^ However, although it is now clear that certain lncRNAs are important to brain development, knowledge fundamental to the understanding of lncRNA molecular mechanism(s) remains limited.

The lncRNA *Pnky* (a.k.a. *lncpnky*, *lncRNA Pou3f2 adjacent neural key regulator*) is a nuclear-enriched, evolutionarily conserved neural lncRNA that regulates mouse NSCs both *in vitro* and *in vivo.*^6,7^ In NSCs cultured from mouse brain, either *Pnky* transcript knockdown (KD) or *Pnky* conditional knockout (*Pnky*-cKO) increases neuronal production by up to 5-fold.^6–8^ *In vivo*, genetic deletion of *Pnky* results in abnormal cortical development related to this phenotype of accelerated neurogenesis,^7^ and *Pnky*-null mice exhibit abnormal learning and behavior.^9^ Furthermore, expression of *Pnky* from a bacterial artificial chromosome (BAC) transgenic insertion (BAC-*Pnky*) rescues the *Pnky* deletion phenotype both *in vitro* and *in vivo* including behavioral abnormality,^7,9^ providing molecular genetic evidence that this lncRNA functions in *trans*. Thus, multiple orthogonal methods indicate that *Pnky* is a potent, *trans-*acting regulator of neurogenesis that is consequential to neurodevelopment and cognition.

A critical knowledge gap in the field of lncRNA biology is how these molecules exert their functions.^10^ Most RNA molecules greater than 100 bp have an inherent propensity to fold into secondary structures that bring the 5′ and 3′ end in close proximity,^11^ and in some cases, RNA secondary (and tertiary) structures directly underlie biological function.^12,13^ For instance, the secondary structure of mRNAs can regulate translation.^14^ Although there are a growing number of reports that describe lncRNA structure and provide evidence for their biological function,^12,15^ lncRNAs can also regulate biology through unstructured RNA regions.^16^ Furthermore, for lncRNA genes that function *in cis*, even the act of transcription—independent of RNA sequence—can activate the expression of neighboring genes.^17–19^ Thus, just as distinguishing *cis*- vs. *trans*-activity is fundamental to the molecular genetic characterization of lncRNA gene function,^19^ a logical next step toward functional understanding is to determine whether RNA transcripts from the gene fold into discrete 3-dimensional (3D) structures.

Currently, it is not possible to reliably predict lncRNA structure from sequence alone.^20^ To overcome this limitation, chemically probing RNA secondary structure generates experimental data that can drive accurate RNA structure modeling.^21–23^ One such method is selective 2′-hydroxyl acylation analyzed by primer extension and mutational profiling (SHAPE-MaP), which has yielded accurate maps of RNA secondary structure in several viral genomes and noncoding RNAs.^24,25^ For insight into RNA tertiary structure, terbium (III) (Tb^+3^) cleavage analyzed by sequencing (Tb-seq) was recently developed as a high-throughput method for probing transcript for regions that constitute tertiary RNA interactions.^26^ For lncRNAs, such maps of tertiary structure have not been reported.

Knowing the structure of lncRNAs as they exist inside the cell is critically important to understanding biological function, since the folded conformation of the lncRNA *in vitro* and *in cellulo* could be very different. Most experimentally determined models of lncRNA structure have been developed from studies of transcripts produced *in vitro.*^27–30^ While it is relatively straightforward to study the secondary structure of RNAs *in vitro*, determining the folded state of the RNA inside the cell is far more challenging,^16,31–37^ particularly for lncRNAs that are of low abundance. Thus, although knowledge of the folded state of lncRNAs *in cellulo* is critically important, there are only a few examples in the current literature, and none have been reported for lncRNAs that regulate brain development.

In this work, we experimentally determined the structure of *Pnky* transcribed *in vitro* and as it exists *in cellulo* in the nucleus of NSCs isolated from the mouse brain. We discovered that *Pnky* is a highly compact lncRNA, organized with regions of intricate secondary structure that have evidence of tertiary interactions. The structure of *in vitro*-transcribed (IVT) *Pnky* and *in cellulo* was remarkably similar. We integrated this knowledge of *Pnky* RNA structure with the complex cellular phenotype of neuronal differentiation by overlaying the phenotypic data from locked nucleic acid (LNA) oligonucleotide-based experimental perturbations that systematically “tile” across the entire *Pnky* transcript. Together, our results characterize *Pnky* as an lncRNA with potent neurodevelopmental function related to its 3D RNA structure and more generally, illustrate how a stepwise, systematic approach to lncRNA gene studies can build foundationally to an understanding of their cellular functions.

## RESULTS

### *Pnky* adopts a homogeneous, compact, and stable conformation

**Figure 1.**
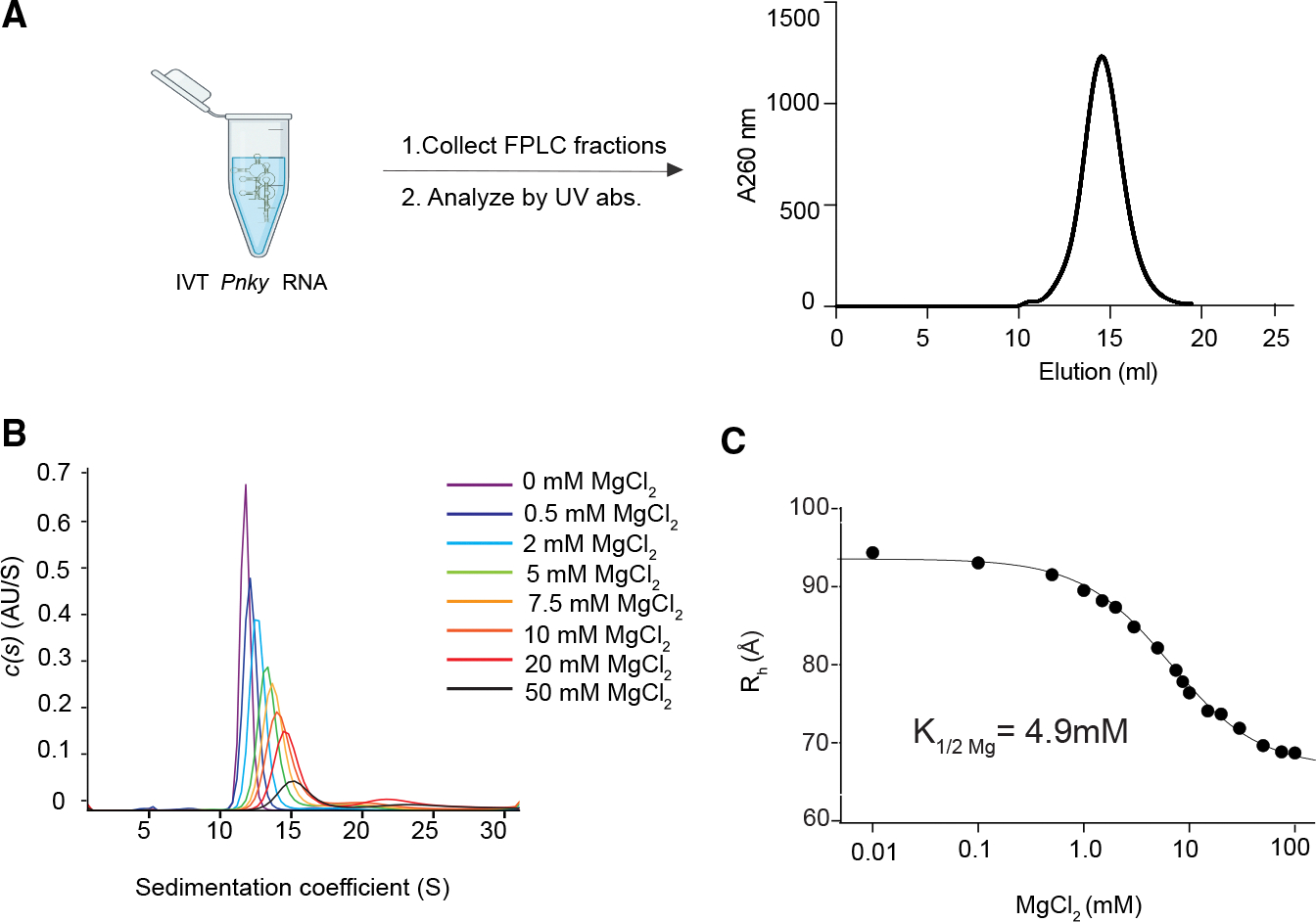
*Pnky* adopts a homogeneous, compact, and stable structure (A) Homogeneity of purified and folded IVT *Pnky* RNA assessed by size-exclusion chromatography. (B) SV-AUC profiles of IVT *Pnky* RNA obtained with increasing concentrations of magnesium. (C) Hill plot of the hydrodynamic radii (R_h_, in angstroms) derived from the SV-AUC experiment. K_1/2 Mg_ indicates the concentration of MgCl_2_ at which 50% of the RNA is folded.

Many functional non-coding RNAs exert biological effects as compact, well-folded molecules.^38–40^ To study the structure of *Pnky* in solution, we used a non-denaturing (or “semi-native”) purification protocol that preserves RNA secondary structure formed during *in vitro* transcription.^41^ With size-exclusion chromatography, IVT *Pnky* eluted a single narrow peak, indicating that this lncRNA exists in solution as a relatively homogeneous population in terms of overall size (Figure 1A).

In RNA structure, magnesium ions (Mg^2+^) play an important role in stabilizing RNA folding by neutralizing negative charges on the phosphate backbone.^42^ We performed sedimentation-velocity analytical ultracentrifugation (SV-AUC) at physiological potassium (K^+^) concentration to monitor the degree of molecular compaction as a function of Mg^2+^ concentration (Figure 1B). In SV-AUC analysis, RNA compaction is observed as an increase in sedimentation velocity and a decrease in UV absorption.^43^ As Mg^2+^ concentration increased, the sedimentation coefficient of *Pnky* increased and UV absorption decreased (Figure 1B), indicating a magnesium-dependent, global compaction of the *Pnky* molecule. Across the wide range of Mg^2+^ concentrations (0–100 mM), *Pnky* remained monodisperse, consistent with this lncRNA adopting a homogeneous, compact structure.

We used SV-AUC data to determine the hydrodynamic radius (R_h_) of *Pnky*. With increasing concentrations of Mg^2+^, *Pnky* RNA compacts with the radius decreasing from ~95Å to 74.4Å. Fitting the R_h_ values at each Mg^2+^ concentration to the Hill equation yielded a K_1/2 Mg_ of 4.9 mM (Figure 1C), a value comparable to that of lncRNA *RepA*/*Xist* (K_1/2 Mg_ 4.8 mM)^44^ and even lower than those of other well-structured non-coding RNAs such as the ai5ɣ group IIB intron (K_1/2 Mg_ 15 mM).^45^ The low K_1/2_ Mg^2+^ for *Pnky* compaction signifies a high degree of structural stability.

### *Pnky* RNA has intricate secondary structure

To experimentally examine the secondary structure of IVT *Pnky*, we used SHAPE-MaP.^24,46^ For SHAPE-MaP, RNA molecules are treated with a hydroxyl-selective electrophile that selectively acylates conformationally flexible nucleotides at the 2′-hydroxyl, resulting in a bulky adduct at single-stranded and/or flexible nucleotides. During MaP readout, the bulky 2′-O-adducts are reported as mutations in the cDNAs produced by reverse transcription (RT).

**Figure 2.**
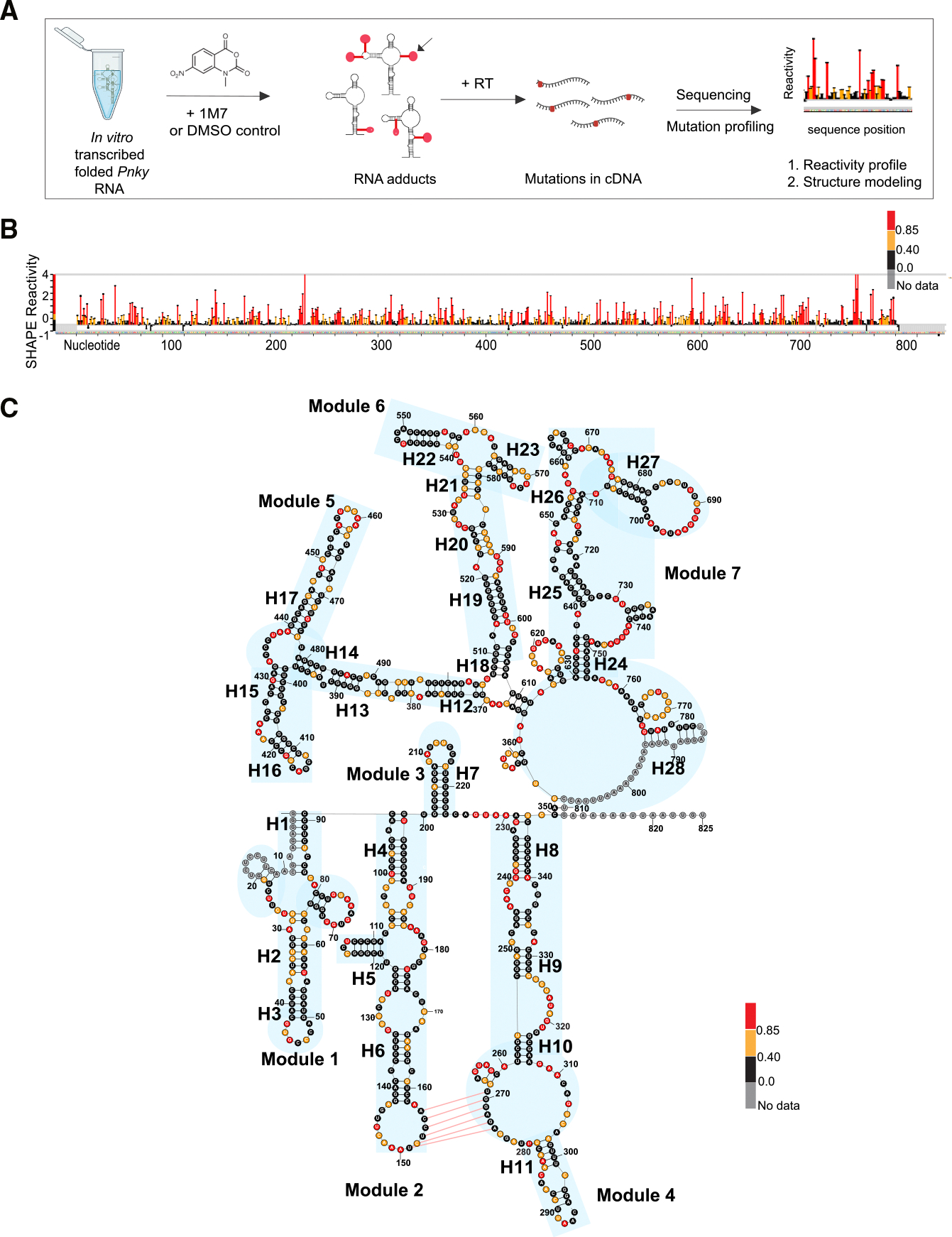
*Pnky* has intricate secondary structure *in vitro* (A) Schematic of the *in vitro* SHAPE MaP of IVT *Pnky* RNA. (B) Representative SHAPE reactivity profile of IVT *Pnky* RNA at nucleotide resolution from *in vitro* SHAPE MaP experiments (*n* = 2). Mean ± SE reactivities are shown where red bars indicate high reactivity (>0.85), yellow bars indicate medium reactivity (0.4–0.84), and black bars indicate low reactivity (<0.4) to SHAPE reagent 1M7. Nucleotides with no data are shown in gray. (C) Minimal free-energy secondary structure model using SHAPE reactivities as constraints. Structural modules are highlighted in blue. Pseudoknot interaction (red lines) predicted using SHAPEKnots between stem loops in modules 2 and 4. See also Figure S1 and S2; Tables S1 and S2.

For *Pnky* SHAPE-MaP, we used 1-methyl-7-nitroisatoic anhydride (1M7) as the electrophile for RNA modification. Based on SV-AUC analysis, we determined that at 15 mM Mg^2+^ (three times the experimentally determined K_1/2 Mg_), *Pnky* is fully folded and monodisperse and thus optimal for structural studies. We treated folded *Pnky* with 1M7 or DMSO, performed MaP-RT, and amplified the resultant cDNA with *Pnky*-specific primers, producing two overlapping amplicons that cover the entire length of the RNA (Figures S1A and S1B) for readout by high-throughput sequencing (Figure 2A).

We performed two independent SHAPE-MaP experiments using independently transcribed, folded preparations of IVT *Pnky* RNA obtained on two separate days. For both 1M7-treated and DMSO control, we obtained two datasets (Figure S2A), each having an average read depth >50,000× and effective reactivity data for 97.8% (763/780) of the nucleotides (Table S1). The relative mutation rates of the 1M7-treated samples were significantly higher than those of the DMSO controls (Figure S2A), with excellent concordance across the replicates (Pearson correlation r = 0.94) (Figure S2B). We used these data to determine SHAPE reactivity at single-nucleotide resolution across the length of *Pnky* (Figures 2B and S2C).

The SHAPE reactivity data were then used as constraints for *de novo* secondary structure prediction.^24^ IVT *Pnky* RNA exhibited highly complex, intricate secondary structure. More than 65% of the nucleotides were involved in base pairing, forming 28 helical segments, 18 terminal loops, 15 internal loops, and 12 junction regions (Figures 2C and S2D), which is comparable to other noncoding RNAs known to be highly structured.^12^

Examination of the secondary structure map revealed seven distinct structured regions that we refer to as the “modules” (highlighted in blue), each formed of multiple helical segments, loops, and junctions. (Figure 2C). Module 1 (nt 1–91) has three helices (H1–H3), one 4-way junction, and three hairpin stem loops. Module 2 (nt 92–200) consists of three helices (H4–H6) with internal loops, a three-way junction, and a terminal hairpin stem loop. Module 3 (nt 201–224) consists of a single helical region (H7) with a hairpin loop. Module 4 (nt 232–350) has 4 helices (H8–H11), a short single-stranded region (nt 225–231), and a three-way junction. Modules 5 and 6 (nt 371–505 and nt 507–608, respectively) each consist of six helical regions (H12–H17 and H18–H23, respectively) that fold into dumbbell-like structures. Both modules 5 and 6 radiate from a short stem that emerges from a wheel-like structure of module 7 (nt 613–774) from which five other substructures containing five helices (H23–H28) emanate.

RNA helices can assemble into a tertiary structure called a “pseudoknot,” which consists of two stem-loop structures in which half of one stem is interposed between the two halves of another stem. RNA pseudoknots play important roles in the expression of many genes.^32,47,48^ Using ShapeKnots,^49^ a pseudoknot was predicted at terminal loops of modules 2 and 4 (indicated by red lines in Figure 2C), with base-pairing interactions across the single-stranded regions of the loops being supported by low SHAPE reactivities at nt 151–156 and 269–274 (Figures 2B and S2C). Furthermore, incorporating the pseudoknot as a hard constraint in structure prediction lowers the Shannon entropy, suggesting that the pseudoknot-containing model of IVT *Pnky* is more thermodynamically stable (Figure S2E).

### Tb-seq detects regions of tertiary structure in *Pnky*

The highly compact, stable, and monodisperse nature of *Pnky* in solution is a strong indication that this lncRNA contains specific tertiary structures that underlie its overall 3D structure. Identifying regions of stable RNA tertiary structure is challenging, which likely explains why very few lncRNAs have been studied at this level.

To form specific tertiary interactions, multiple RNA secondary structures must bring their negatively charged phosphate backbones into proximity. Such interactions are often stabilized by the local coordination of multivalent cations such as Mg^2+^, which neutralize local negative charge. Lanthanide ions, such as terbium (III) (Tb^3+^), can take the place of Mg^2+^ within an RNA structure, but due to its chemical nature, Tb^3+^ induces local RNA cleavage at the site of binding.^42,50^ Recently, a high-throughput sequencing method, Tb-seq, was developed to identify sites of Tb^3+^ cleavage, systematically identifying sites of tertiary interactions within an RNA molecule.^26^

**Figure 3.**
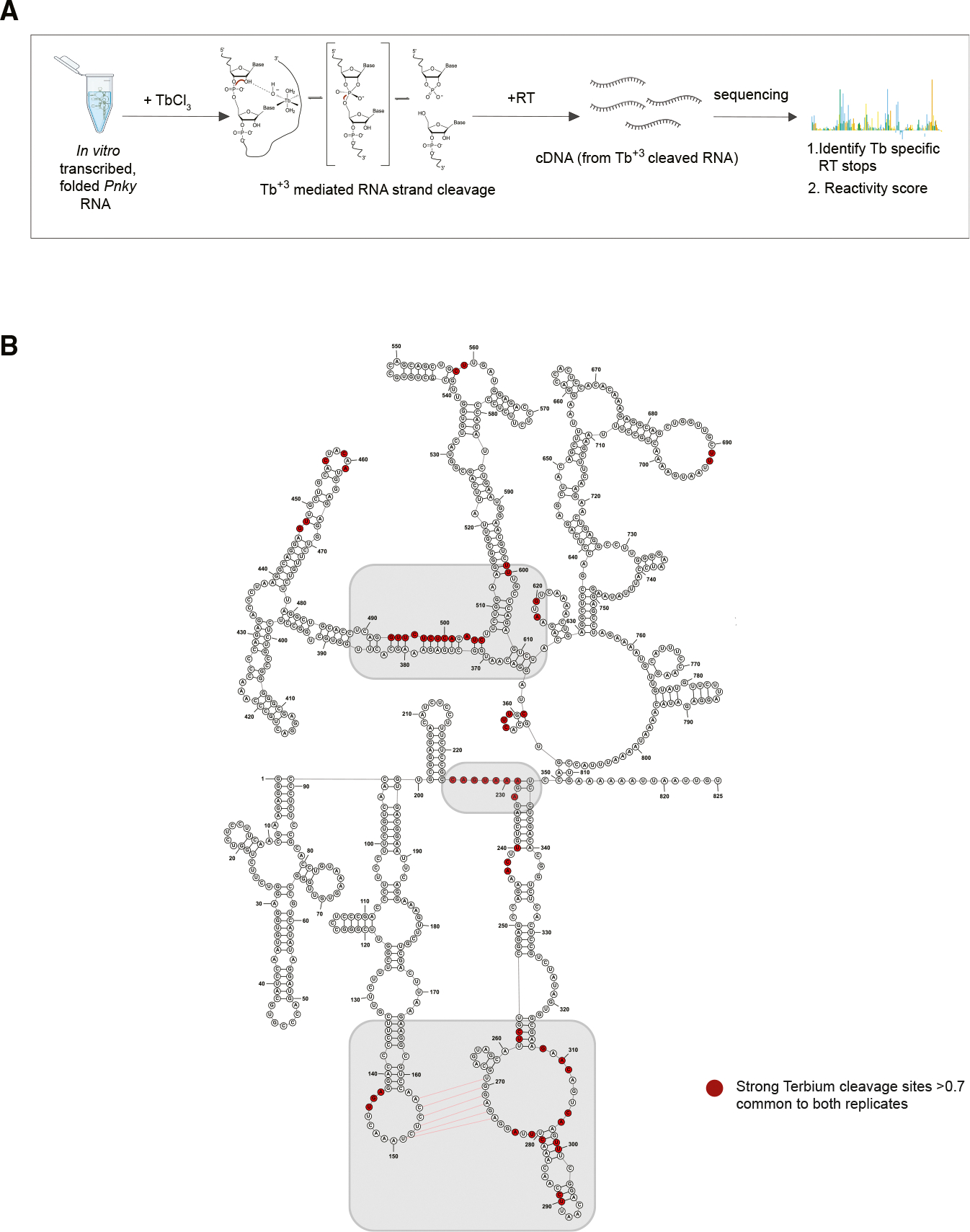
Tb-seq detects regions of tertiary structure in *Pnky* (A) Schematic showing terbium-seq workflow. (B) Secondary structure of IVT *Pnky* RNA displaying sites of strong Tb^3+^ cleavage (red) with three regions of concentrated Tb reactivity highlighted in gray. See also Figure S3.

We used Tb-seq to study the tertiary structure of *Pnky*. Briefly, after treating IVT, folded *Pnky* with TbCl_3_, we performed RT, and sites of strand cleavage were identified with high-throughput sequencing (Figure 3A). For each nucleotide, we determined the probability of Tb^3+^-dependent cleavage vs. spontaneous cleavage, generating a reactivity score (see STAR Methods) (Figure S3A). The reactivity scores across two independent replicates were highly similar (Pearson correlation r = 0.97) (Figure S3B).

To identify sites that likely result from specific, site-bound Tb^3+^-dependent cleavage, we used two criteria. First, a stringent reactivity value of >0.7 was used as a threshold for each site. Second, these sites must be observed in two independent replicates. Nucleotide sites that satisfy these criteria are highlighted in red in the secondary structure diagram of *Pnky* (Figures 3B and S3C).

*Pnky* had 57 sites of Tb^3+^ induced cleavage. The distribution of most (70.2%) Tb^3+^ cleavage sites was concentrated in three major regions of the *Pnky* secondary structure model (Figure 3B). One region corresponds to the predicted pseudoknot that crosses modules 2 and 4, with Tb^3+^ reactivity prominent around the “kissing loop” component (Figure 3B, highlighted in gray). The second major region is located at the boundary between modules 3 and 4 (Figure 3B, gray box). The third region of Tb^3+^ reactivity is found among the nucleotides of modules 5, 6, and 7 that map to secondary structures that are proximal to their emanation from the major ring of module 7 (Figure 3B, gray box). In addition to these three clusters of Tb^3+^ reactivity, a minor number of sites (29.8%) are found among nucleotides of distal aspects of secondary structures of modules 5, 6, and 7. Together, these data represent the first systematic analysis of tertiary structure in an lncRNA and for *Pnky*, provides evidence for it having tertiary interactions.

### *Pnky* structure *in cellulo* is similar to its *in vitro* folded state

In addition to having a high-quality structural map of IVT *Pnky*, to understand the potential relationship between its structure and biological function, it is important to also obtain a map that captures the conformational states and potential RNA “protections” of *Pnky in cellulo*. Such knowledge of *Pnky* structure *in cellulo* is important to the interpretation of experiments that interrogate the biological function of specific regions of the lncRNA.

SHAPE-MaP can be performed *in cellulo* by treating cells with the electrophile reagent, extracting the RNA, then performing RT and downstream sequencing analysis.^51^ For RNAs that are abundant, such as the genome of RNA viruses, in-cell SHAPE-MaP has provided data of sufficient depth for *de novo* structural modeling.^25^ However, for RNAs that are at low abundance, such as most lncRNAs, *in cellulo* SHAPE-MaP reactivity data have been mostly used to corroborate selected regions of the *in vitro* conformation, not to generate an entirely structural model *de novo.*

We sought to perform SHAPE-Map for *de novo* secondary structural modeling of *Pnky* as it exists in the cellular nucleus of primary neural stem cells that exhibit a strong *Pnky*-dependent phenotype rather than in the immortalized cell lines. Given our prior results, we selected NSC cultures established from the postnatal ventricular subventricular zone (V-SVZ) for our *in vivo* SHAPE-MaP studies.^6,7^

*Pnky* is a nuclear-enriched lncRNA that is at relatively low abundance (~10–20 copies per nucleus), which presents a nontrivial technical challenge. For in-cell SHAPE-MaP to be successful, it is necessary to acylate the target RNA inside the cell, which is far less efficient than for purified RNA *in vitro*, and the low concentration of *Pnky* necessitates the use of relatively large numbers of cells cultured from a specific part of the mouse brain. In early experiments, we used 70–90 million V-SVZ NSCs cultured from postnatal day 7 (P7) *Pnky*^*+/+*^ mice for in-cell SHAPE-MaP; however, SHAPE reactivity data were not sufficient for reliable *de novo* structural modeling.

Increasing RNA transcript abundance can improve in-cell SHAPE-MaP studies.^52^ Although it is possible to express *Pnky* at very high levels from lentiviral vectors,^53^ we have found that vector-expressed *Pnky* transcripts localize primarily in the cytoplasm (unpublished observations), unlike the normal nuclear localization of *Pnky*.^6^ In contrast, BAC-expressed *Pnky*—which rescues *Pnky*-KO phenotypes including at the level of the transcriptome—remains nuclear enriched.^7^ Importantly, the levels of *Pnky* in V-SVZ NSC cultures from *Pnky*^+/+^; BAC-*Pnky* mice were only ~5- to 10-fold higher (~100 copies per nucleus) than those from *Pnky*^+/+^ mice.

**Figure 4.**
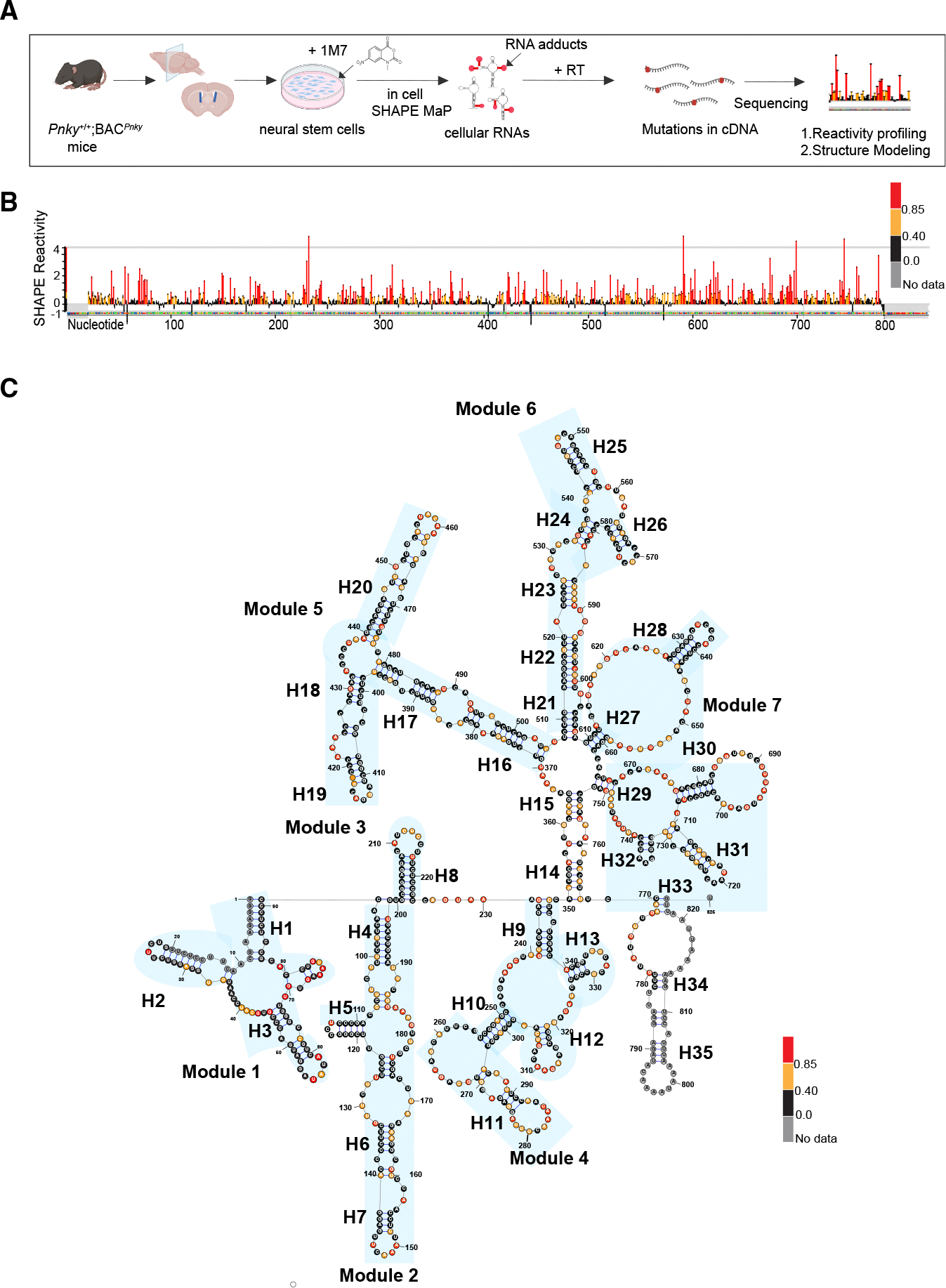
*Pnky* structure *in cellulo* is similar to its *in vitro* folded state (A) Schematic of the in-cell SHAPE MaP for *Pnky* RNA in primary culture of mouse neural stem cells. (B) Representative *in cellulo* SHAPE reactivity profile for *Pnky* RNA at nucleotide resolution obtained from in-cell SHAPE probing experiments in neural stem cells (*n* = 2). Mean reactivities (±SE) are plotted where red bars indicate high reactivity (>0.85), yellow bars indicate medium reactivity (0.4–0.84), and black bars indicate low reactivity (<0.4) to SHAPE reagent 1M7. Nucleotides with no data are shown in gray. (C) Minimal free-energy secondary structure model using in-cell SHAPE reactivities at nucleotide resolution for *Pnky* RNA. Blue shaded boxes demarcate the modules of the *in cellulo* structural model. See also Figure S4; Tables S1 and S2.

Thus, for *Pnky* in-cell SHAPE-MaP analysis, we used NSC cultures established from the V-SVZ of P7 *Pnky*^+/+^; BAC-*Pnky* mice. For each biological replicate, we established primary V-SVZ NSC cultures from multiple P7 *Pnky*^+/+^; BAC-*Pnky* litters, treated 70–90 million NSCs with either 1M7 (50mM) or DMSO, isolated the nuclei, extracted nuclear RNA, and then depleted ribosomal RNA to enrich for *Pnky* RNA (Figure 4A). After RT, PCR amplification with *Pnky*-specific primers produced two overlapping amplicons of expected size (Figure S4A), generating sequencing libraries that cover the entirety of the 825-nt transcript. To the best of our knowledge, this is the first in-cell SHAPE data for a neural RNA from the primary cell culture of neural stem cells.

Across the two replicates, we obtained effective reactivity data for 97.8% of the nucleotides (Table S1) and significantly higher mutation rates with 1M7 as compared to DMSO control, indicating successful modification of the RNA *in cellulo* (Figure S4B). Furthermore, SHAPE reactivities from both replicates had strong correlation across the entire *Pnky* transcript (Pearson correlation r = 0.84) (Figures 4B, S4C, and S4D).

We then used in-cell SHAPE reactivities to experimentally constrain secondary structure modeling.^24^ Nearly 70% of the nucleotides were involved in base pairing, forming 35 helical segments, 18 terminal loops, 18 internal loops, and 10 junction regions (Figures 4C and S4E), which is similar to the degree of structure observed for IVT *Pnky* (Figure 2C). Thus, like its folded state *in vitro, Pnky in cellulo* is highly structured with a similar distribution of RNA structural motifs.

Like the conformation of IVT *Pnky* (Figure 2C), the secondary structure of *in cellulo Pnky* can be divided into seven modules (Figure 4C). Across the two structural models, the number of nucleotides within each module is very similar, and the borders between each module are within a few nucleotides of each other (Table S2). We note that two modules (modules 4 and 7) appear different *in cellulo* as compared to *in vitro*. Module 4 has different secondary structures across the two models, and the pseudoknot interaction across modules 4 and 2 is not predicted *in cellulo*. Differences in module 7 are likely a result of suboptimal modeling due to the local binding of the primers used for reverse transcription and the consequent lack of SHAPE reactivity data for the nucleotides near the 3′ end.

Five of the seven modules of *Pnky* are remarkably similar across the *in cellulo* and *in vitro* models. For example, module 1 can be characterized by an internal bulge from which three hairpin loops emerge. Module 2 consists of a helix followed by an internal bulge, followed by a bulge with a side-protruding hairpin loop, then another bulge from which another structure emerges that ends in a terminal hairpin loop. Module 3 is nearly identical, being defined by a solitary hairpin loop. Modules 5 and 6 are dumbbell shaped and have highly similar patterns of helices, internal bulges, and hairpin loops. Thus, these models of *Pnky* structure *in cellulo* and *in vitro*—both of which were developed *de novo* and independent of one another—exhibit remarkable similarity.

### Specific LNA ASOs that target *Pnky* can phenocopy *Pnky*-KD

Disrupting RNA secondary structures with LNA antisense oligonucleotides (ASOs) is a powerful approach for determining if observed RNA structures are functional.^54,55^ LNAs are non-natural RNA base analogs that increase the melting temperature (T_m_) of nucleoside duplexes, which allows LNA ASOs to outcompete endogenous RNA-RNA duplexes that comprise secondary structure.^56^ Because LNAs have non-natural chemical structure, the binding of LNA ASOs does not activate RNase H-mediated degradation of the RNA target.^56^ Thus, LNA ASOs can reveal lncRNA function related to RNA structure and not simply lncRNA transcript abundance.

**Figure 5.**
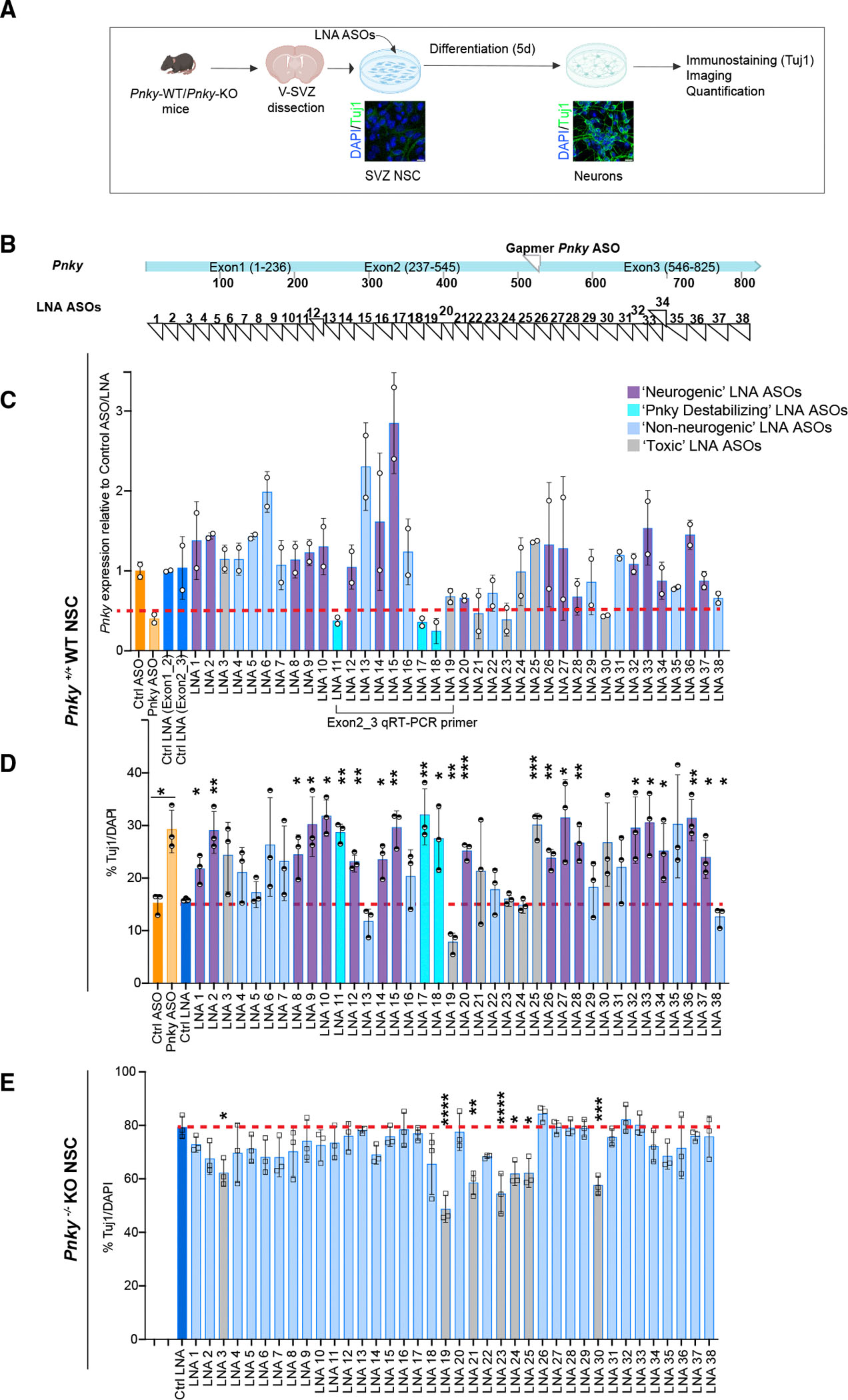
Specific LNA ASOs that target *Pnky* can phenocopy *Pnky*-KD (A) Schematic of the LNA ASO and gapmer ASO transfection in primary NSCs and neurogenesis assay. (B) Location of LNA and gapmer ASO binding sites on the *Pnky* RNA. (C) Relative fold change in *Pnky* transcript levels in *Pnky-*WT V-SVZ cultures by RT-qPCR 21–24 h after transfection with LNA and gapmer ASOs. (D) Tuj1 ICC in 5 days (5d) differentiated V-SVZ cultures established from *Pnky*-WT mice. Quantification using % Tuj1+ normalized to DAPI-positive cells in *Pnky*-WT NSCs. (E) Tuj1 ICC in 5d differentiated V-SVZ cultures established from *Pnky*-KO mice. Quantification using % Tuj1+ normalized to DAPI-positive cells in *Pnky*-KO NSCs. *p < 0.05, **p < 0.01, ***p < 0.001, ****p < 0.0001; ns, non-significant. Data represented as mean ± SD for *n* = 3 independent technical replicates of V-SVZ culture obtained from pooled dissection of postnatal day 7 animals. Two-tailed unpaired *t* test for WT dataset and ordinary one-way ANOVA with multiple comparisons for the KO dataset. See also Figures S5 and S6; Tables S3.

To evaluate the *Pnky*-targeting LNA ASOs for function in neural development, we used the well-characterized primary V-SVZ NSC culture system that recapitulates key aspects of neurogenesis *in vivo.*^57–59^ V-SVZ NSC cultures can be passaged as a readily transfectable monolayer, and neuronal differentiation is induced by removal of mitogenic factors (Figure 5A). In V-SVZ NSC cultures, *Pnky* transcript KD or *Pnky* cKO has a “positive” phenotype, increasing neurogenesis.^6,7^

We used LNA ASOs to interrogate the length of the *Pnky* transcript for biological function. Instead of targeting selected *Pnky* secondary structures predicted by SHAPE-MaP analyses, we chose to pursue an unbiased approach by designing a set of 38 LNA ASOs that tile the entire *Pnky* lncRNA (Figures 5B; Table S3). Establishing a rigorous and scalable neuronal differentiation assay for LNA ASO studies is challenging given the developmental complexity of neurogenesis, which can be adversely affected by experimental interventions such as transfection. To benchmark the assay and illustrate the positive neurogenic phenotype of *Pnky*-KD in LNA transfection studies, we produced an LNA ASO with a “gapmer” design, wherein the central nucleotides of the oligonucleotide are chemically unmodified DNA, which allows RNase H-mediated degradation of the RNA target.^54,56^ One day after transfection, the *Pnky* gapmer ASO reduced *Pnky* transcript levels by ~55% as compared to non-targeting gapmer control (Figure 5C, orange bars), and after 5 days of differentiation, gapmer-mediated *Pnky*-KD increased neurogenesis by nearly 2-fold (Figure 5D, orange bars), as expected.

We assayed in parallel all 38 LNA ASOs alongside gapmer ASO and control oligonucleotides in triplicate NSC cultures from *Pnky* wild-type (WT) mice to assess the neuronal production phenotype (Figures 5D, S5A, and S5B). We also assayed for the effect of these LNA ASO transfections on *Pnky* transcript abundance by quantitative reverse-transcription PCR (RT-qPCR) (Figure 5C). As compared to transfection of LNA oligonucleotide control (Figure 5D, dark blue bar), 22 LNA ASOs that target *Pnky* increased neuronal production by 1.5- to 2-fold (Figure 5D, purple bars and a subset of gray and teal bars) and one LNA ASO reduced neurogenesis by 0.9-fold (Figure 5D, LNA 38). Thus, overall, 60% (23 of 38) of the LNA ASOs produced a neurogenic phenotype, and of these, the vast majority (95.6%, 22 of 23) phenocopied the effect of *Pnky*-KD.

To filter for LNA ASOs with potential off-target effects, we also assayed in parallel all 38 LNA ASOs in NSC cultures from *Pnky*-KO mice (Figures 5E, S6A, and S6B). None of the LNA ASOs increased neurogenesis from NSCs that lack *Pnky* transcript. However, we did identify 7 LNA ASOs that reduced neuronal production in *Pnky*-KO NSCs (Figure 5E, gray bars). Thus, while 7 LNA ASOs had a *Pnky*-independent negative effect upon neurogenesis, none of the other 31 LNA ASOs had evidence of an off-target positive neurogenic phenotype.

Although LNA ASOs do not directly induce degradation of the RNA target, loss of the RNA target could still be observed after transfection. We, therefore, next filtered for LNA ASOs that reduced *Pnky* levels after transfection, since such a change would confound the interpretation of a neurogenic phenotype and its potential relationship to RNA structure. Among the 31 LNA ASOs without evidence of off-target effect, 3 reduced the detection of *Pnky* to less than 50% of control (Figure 5B, LNAs 11, 17, and 18). Of note, these 3 LNA ASOs also increased neurogenesis by 1.5- to 2.1-fold (Figure 5C, teal bars), similar to the phenotype observed with gapmer-mediated *Pnky*-KD (Figure 5C, orange bars).

We next examined the 28 LNA ASOs that did not reduce *Pnky* transcript levels or have off-target effects. In this filtered subset, 39% (11 of 28) of the LNA ASOs did not increase neurogenesis (Figure 5D, light blue bars), indicating that the transfection of *Pnky*-targeted LNA ASOs does not generally promote neurogenesis. Importantly, however, 60.7% (17 of 28) of the LNA ASOs increased neuronal production by 1.5- to 2.1-fold (Figure 5D, purple bars). The identification of multiple *Pnky*-targeted LNA ASOs that increase neurogenesis without decreasing *Pnky* transcript levels is consistent with a model in which RNA structure and/or interaction with proteins underlies the function of this neurodevelopmental lncRNA.

### LNA ASO phenotypic data map to specific structured regions of *Pnky*

**Figure 6.**
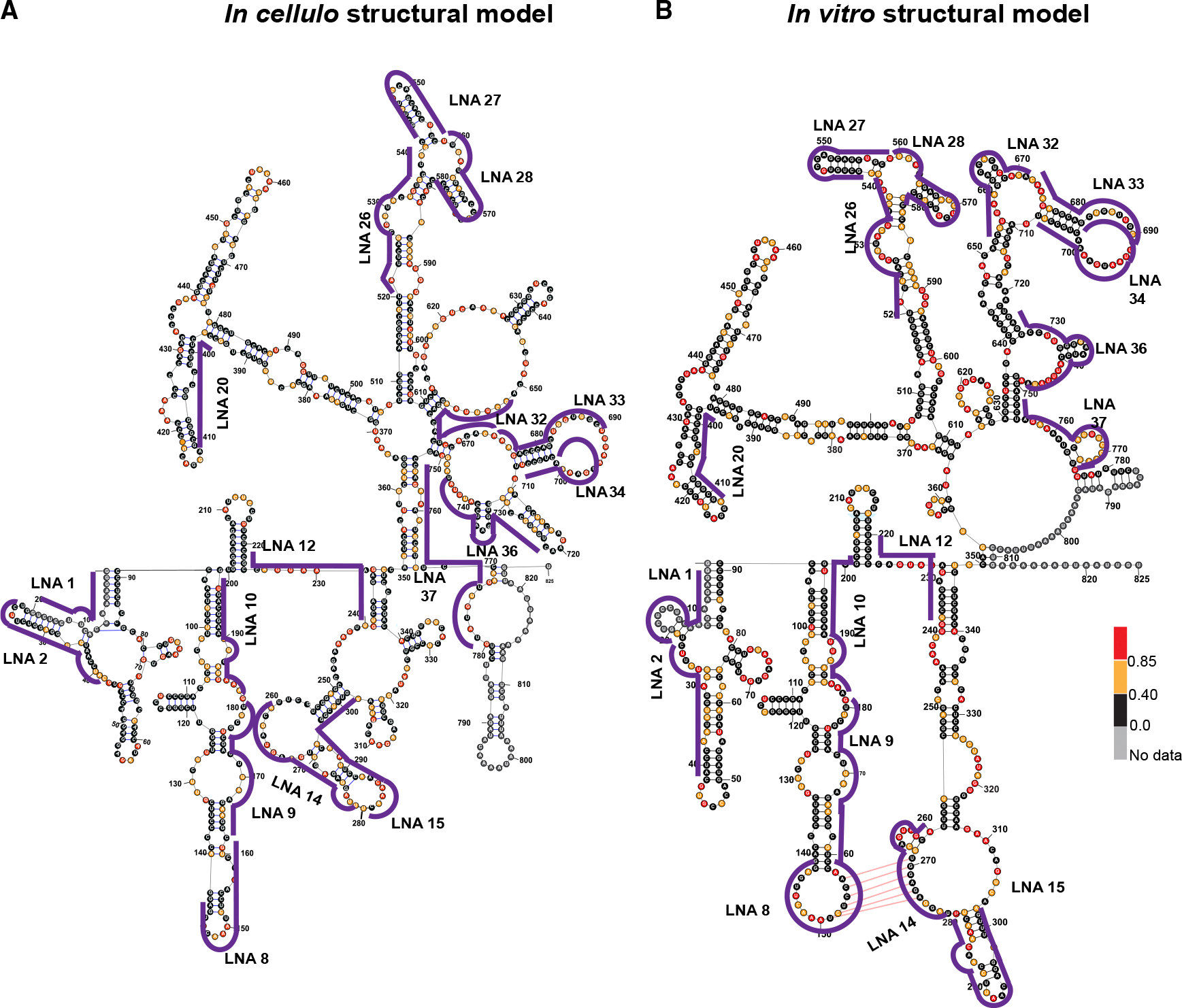
LNA ASO phenotypic data maps to specific structured regions of *Pnky* (A) Locations of the neurogenic LNA ASOs (purple) mapped onto the *in cellulo* structural model of *Pnky* RNA. (B) Locations of the neurogenic LNA ASOs (purple) mapped onto the structural model of IVT *Pnky* RNA. See also Table S3.

**Figure 7.**
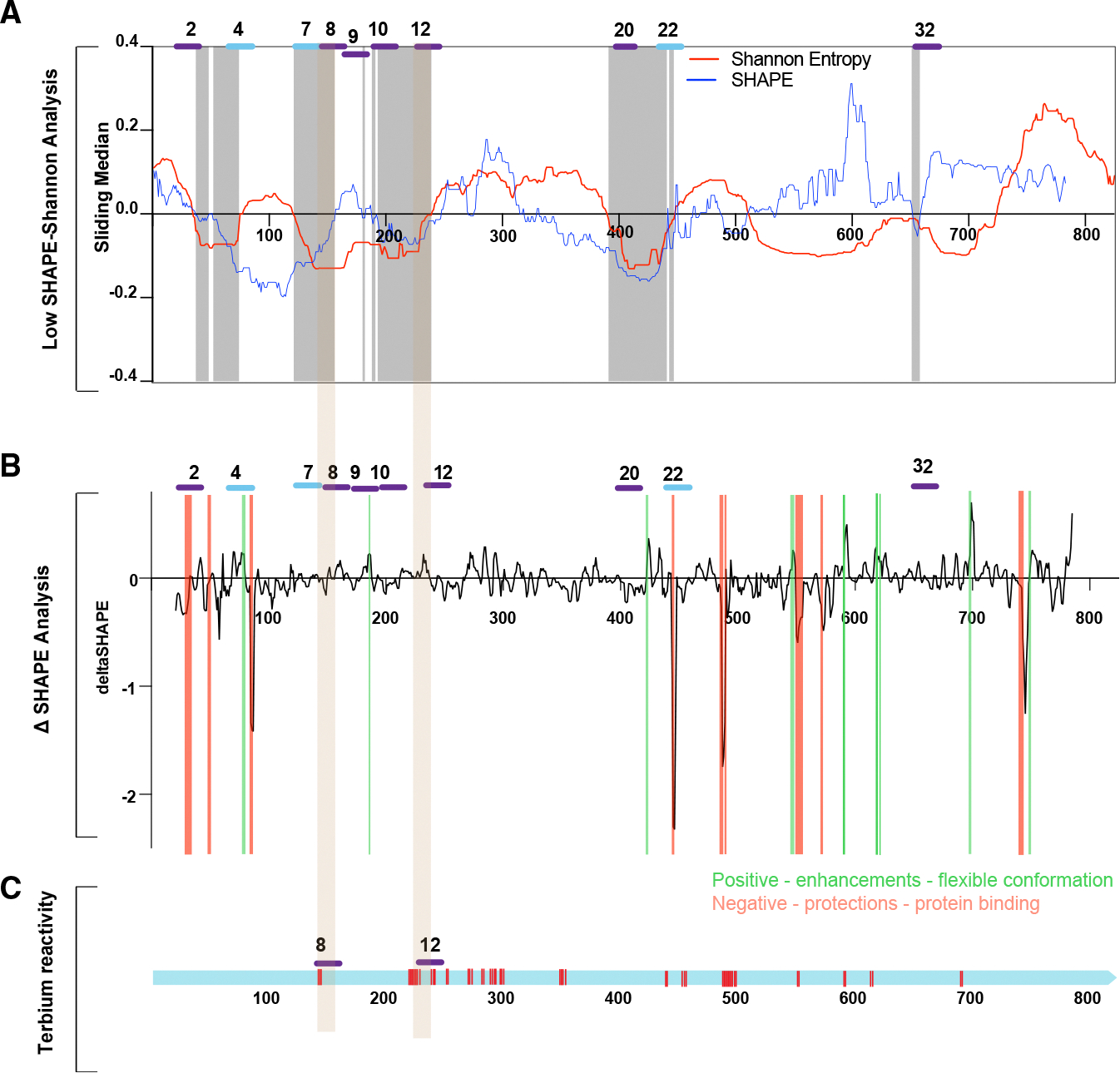
Neurogenic LNA ASOs target structured regions in the *Pnky* RNA (A) SHAPE-Shannon entropy plot with low SHAPE reactivity-low Shannon entropy (lowSS- structured) regions highlighted in gray. Colored bars along the top indicate the location of the 'Neurogenic' (purple) and 'Non-neurogenic' LNA ASOs (light blue) that overlap with these lowSS regions. (B) ΔSHAPE plot of *Pnky*. Regions with significant positive ΔSHAPE scores (flexible conformation *in cellulo*) are highlighted in green; regions with significant negative ΔSHAPE scores (protected *in cellulo*) are highlighted in orange. Colored bars along the top indicate the location of 'Neurogenic' (purple) LNA ASOs and 'Non-neurogenic' LNA ASOs (light blue). (C) Plot of terbium-reactive nucleotides (vertical red bars) along the length of *Pnky*. Purple bars along the top indicate the location of the neurogenic LNA ASOs that overlapped terbium-reactive nucleotides and also overlapped with lowSS regions (in A, above) but did not overlap with deltaSHAPE protections (in B, above). See also Table S4.

We mapped the locations of the 17 LNA ASOs that increased neurogenesis without decreasing *Pnky* transcript levels (mapped in purple in Figures 6A, 6B, and S7) onto the *in cellulo* and the *in vitro* structural models. To investigate whether the neurogenesis phenotype observed with these 17 LNA ASOs is potentially related to their disruption of RNA structure, we first identified regions of *Pnky* that have the most evidence for being “highly structured” by performing low SHAPE/Shannon entropy (lowSS) analysis, which identifies regions that have concomitant low nucleotide flexibility and local thermodynamic stability. *Pnky* had 9 regions with lowSS (gray columns, Figure 7A). Among the 17 LNA ASOs that had a positive neurogenic phenotype, 7 (41%) overlapped with lowSS regions (purple bars, Figure 7A). In contrast, only 3 of the 11 (27%) LNA ASOs that lacked a neurogenic phenotype overlapped with the lowSS regions (light blue bars, Figure 7A).

Next, we performed ΔSHAPE analyses,^60^ which quantitatively assesses the nucleotide reactivity differences between the in-cell and *in vitro* SHAPE-MaP data. For each nucleotide of *Pnky*, ΔSHAPE (average 1M7 reactivity difference between *in vitro* and in-cell SHAPE-MaP score) was used to calculate a *Z* score to identify regions of significant (<0.05) reactivity differences. Overall, most nucleotides (92.8%, 766 of 825 nt) exhibited non-significant reactivity differences between the in-cell and *in vitro* SHAPE MaP data (Figure 7B), which corresponds to the similarity of the secondary structural models. Importantly, SHAPE analysis revealed 9 small regions of significant “negative” ΔSHAPE scores (orange columns, Figure 7B; Table S4), which may represent protections due to protein binding *in cellulo.* Nine regions (green columns, Figure 7B; Table S4) had significant positive ΔSHAPE scores, which may represent regions with more flexible conformation in the cell. Of the 7 LNA ASOs that target lowSS regions, only LNA 2 partially overlapped with one of the 9 regions with negative ΔSHAPE scores (Figure 7B). Thus, LNAs 8, 9, 10, 12, 20, and 32 generate a positive neurogenic phenotype without targeting regions that have evidence of protein binding *in cellulo*.

Finally, we used the Tb-seq analyses to augment our inference of RNA structure as it relates to LNA phenotype and targeting location (Figure 7C). Notably, of the 7 neurogenic LNAs that target lowSS regions (Figure 7A), LNAs 8 and 12 also target regions with evidence of tertiary interactions (terbium reactivity = light orange columns, Figure 7C) without overlapping regions of protection *in cellulo* (Figure 7B). Overall, the integration of RNA structural maps with a systematic approach to LNA targeting provides the groundwork from which a structure-function-based mechanistic understanding of *Pnky* can be further built.

## DISCUSSION

For the growing number of individual lncRNAs that have been shown to regulate neurodevelopment, the molecular underpinning of their function is poorly understood.^5,61^ For the investigation of *Pnky*, we have taken a stepwise, systematic approach to understanding its function in neurogenesis, which has led us to the RNA structure studies presented here.

IVT *Pnky* RNA exhibited a homogeneous, compact structure with remarkable stability. The stability of *Pnky* folding, as assessed by K_1/2 Mg_, is comparable to the RepA region of *XIST* and even lower than that of ai5ɣ group IIB intron, both being examples of RNA structure that appears to play a role in their cellular function.^44,45^ The biophysical stability of *Pnky in vitro* adds importance to the hypothesis that this lncRNA transcript possesses biological information in its 3D structure.

Underlying the stable global conformation of *Pnky in vitro* was an intricate secondary structure composed of seven modules. Of note, we use the term modules simply to describe ensembles of secondary structural features. In contrast to previous studies in which truncated constructs are created and studied biophysically *in vitro,*^27,44^ we do not claim that the modules of *Pnky* represent autonomous folding domains that can function in isolation. Other studies of IVT lncRNAs have indicated a biophysically modular nature of lncRNA secondary structure. For example, *HOTAIR* and the *RepA* region of XIST autonomously fold into modular domains that facilitate interaction with chromatin regulators.^27,31,62^ The lncRNA *Braveheart* possesses a G-rich internal loop that physically interacts with a specific transcription factor critical for cardiomyocyte differentiation,^29^ but this region was not determined to be autonomously modular in nature. Whether *Pnky* consists of autonomously folding modules remains to be determined.

We performed SHAPE-MaP in primary NSCs, which is significant because it demonstrates the feasibility of studying lncRNA secondary structure in normal cells (i.e., not cancer cell lines) with complex developmental phenotypes. The secondary structure of *Pnky* was modeled to be very similar *in vitro* as compared to *in cellulo*. This similarity suggests that the overall structure of *Pnky* is not dramatically shaped by interactions with nuclear proteins or other molecules *in cellulo*, or that the conformational changes are too transient to be captured by averaged reactivity profiles inherent to the SHAPE-MaP technique.

There is very little knowledge about lncRNA tertiary structure. For a very limited number of lncRNAs, techniques such as atomic force microscopy (for *Meg3*)^32^ and small-angle X-ray scattering and NMR (for *Bvht*^30^ and *XIST*^63,64^) have been useful to gain an overview of the higher-order folding. Tb-seq was developed as a comparatively high-throughput method of identifying nucleotides involved in the compaction of RNA into tertiary structures,^26^ and Tb-seq analysis of *Pnky* characterized this lncRNA as one with discrete tertiary interactions.

Studies of lncRNA structure can benefit from comparisons to orthologs, and *Pnky* is an evolutionarily conserved lncRNA gene.^6^ For this study, we did not use the strategy of evolutionary comparison because in mice, *Pnky* has been characterized as a fully spliced transcript, whereas the human *PNKY* transcript contains introns between the three conserved exons.^6^ The biological implication of this difference in the linear structure of this lncRNA across these two species is not yet clear, and there are currently no reports of the function of *PNKY* in human NSCs. Furthermore, we did not detect *Pnky* amplicons larger than the expected size in our *in cellulo* SHAPE-MaP studies, although it remains possible that isoforms similar to human *PNKY* exist in the mouse.

For lncRNAs, there is currently very little information to guide the design of structure-function studies. Instead of selecting a handful of structures identified in our models of *Pnky* structure for functional studies, we implemented a strategy that interrogated the entire *Pnky* transcript at approximately 20-nt intervals. For this, we used LNA ASOs, which due to their high Tm (which would outcompete RNA-RNA duplexes) have been found to be structure disrupting in studies of viral genomes.^65–68^ Whether LNA ASOs would be useful for studies of a complex developmental phenotype was unclear.

We studied the role of *Pnky* in neuronal differentiation, which is a complex cellular process that can be easily inhibited (negative phenotype) by experimental perturbations. We reasoned that the observation of increased neurogenesis (positive phenotype) in this assay coupled with the systematic, relatively unbiased approach of LNA-targeted perturbations would provide the experimental scale and robustness to (1) infer a structural basis to *Pnky* function in neurogenesis and (2) prioritize specific RNA regions and/or structures for future mechanistic studies in NSCs.

In our studies conducted in primary mouse NSC cultures, the positive neurogenic phenotype of 17 LNA ASOs supports the hypothesis that the structure of *Pnky*—independent of the level of the lncRNA transcript—plays a critical role in the regulation of neuronal production. An important consideration is whether these LNA ASOs achieve their neurogenic phenotype primarily through structure disruption and/or the potential blocking of interaction(s) with other macromolecules such as proteins. Based on our lowSS, ΔSHAPE, and Tb-seq analyses, at least a subset of LNA ASOs have a positive neurogenic phenotype that appears related to structure disruption and not direct interference with protein binding. Neurogenic LNA ASOs (LNAs 2, 27, 28 and 36, Table S4) also targeted ΔSHAPE-protected regions, suggesting that these may function, in part, by disrupting interactions with nuclear proteins. Although these results do not definitively distinguish structure disruption from the potential steric blocking of macromolecular interactions, the combination of these three analyses help prioritize and categorize specific regions of *Pnky* for more detailed, hypothesis-driven mechanistic studies.

Our discovery that the lncRNA *Pnky* is a compact and highly structured transcript lays important groundwork for understanding how this lncRNA can function in *trans* to regulate neurogenesis. Mapping the locations of the LNAs and their phenotype to both *in vitro* and *in vivo* conformations guide the next steps for experimental investigations into the structure-driven molecular mechanisms of *Pnky.* More broadly, the approaches described for the study of *Pnky* provide an experimental framework for studying structure-function relationships of generally low-abundant lncRNAs in functionally relevant, complex biological contexts.

### Limitations of the study

We did not directly demonstrate changes to *Pnky* structure by LNA ASO binding. Tb-seq is not possible in live cells, and the amount of *Pnky* RNA that can be isolated from primary NSCs is at the limit of detection. Thus, Tb-seq and SHAPE-MaP studies after LNA transfection are not currently possible. Even studies of IVT *Pnky* bound to LNA ASOs would be difficult to interpret, since LNA ASO binding would cause loss of SHAPE reactivity and Tb-induced cleavage for the LNA ASO-paired nucleotides, regardless of potential changes to the secondary/tertiary structure, and this change alone would result in differences in SuperFold modeling. Second, we did not identify specific structural elements of *Pnky* RNA that interact with proteins or nucleic acid partners, which would provide greater insight into how *Pnky* regulates neurogenesis. However, such detailed mechanistic dissection is beyond the scope of this study.

## RESOURCE AVAILABILITY

### Lead contact

Further information and requests for resources and reagents should be directed to and will be fulfilled by the lead contact, Daniel Lim (daniel.lim@ucsf.edu).

### Materials availability

Plasmid and mouse lines generated in this study will be made available upon request.

### Data and code availability

- SHAPE-MaP and terbium-seq data have been deposited in the NCBI SRA database under the identifier BioProject ID: PRJNA1263515.
- This paper does not report original code.
- Any additional information required to reanalyze the data reported in this paper is available from the lead contact upon request.

## STAR★METHODS

### EXPERIMENTAL MODEL AND STUDY PARTICIPANT DETAILS

#### Mus Musculus

All animal related research protocols comply with the relevant ethical regulations approved by the University of California, San Francisco Institutional Animal Care and Use Committee. Animals used in this study were maintained in the University of California, San Francisco Laboratory Animal Resource Center under approved protocol. C57BL/6J mice at postnatal day 7 were used for generation of primary cell culture. Mice of both sexes were used. Therefore, potential sex differences were not assessed in this study, which represents a limitation. Mice were group-housed in cages with sterile bedding and *ad libitum* food and water. Standard conditions of 12-h dark/light cycle, humidity (30–70%) and temperature (20°C–26°C) were maintained. Mice of both sexes were used for all experiments and were used for derivation of primary cell culture at postnatal day 7. Mouse strains used in this study are *Pnky* ^+/+^ (WT), *Pnky*^−/−^(knockout) and *Pnky*^+/+^; BAC-*Pnky* (overexpression). All animals were genotyped by PCR for *Pnky*^+^, and *Pnky*^−^ alleles using the following primers: *Pnky* GT Forward and Reverse 1 and 2 primers. Reaction products: 120 bp (*Pnky*-KO), 221 bp (*Pnky*-WT). Since BAC-*Pnky* contains unaltered *Pnky*, this will also produce the 221-bp product. Primers for BAC-*Pnky* produce a 416 bp reaction product and no amplification in the absence of BAC-*Pnky.*

#### Primary cell culture

All cells were grown in humidified incubators at 37°C in 5% CO2. Cell lines were authenticated for genotype by PCR as described above. The cells were not tested for mycoplasma contamination as these primary cell lines were grown for limited number of passages (5–6).

#### Ventricular subventricular zone (V-SVZ) neural stem cell culture

The brains of P7 mice were dissected out of the skull, and then a coronal slab of approximately 0.5 mm in thickness was obtained manually. Dissections were performed in ice-cold L15 medium to collect the V-SVZ region along the lateral walls of the lateral ventricles. The tissue was dissociated with 300 mL of 0.25% trypsin-EDTA with occasional agitation for 20 min at 37°C, and then the trypsin was quenched with 600 mL of N5 growth medium (see below). Cells were mechanically dissociated by trituration and then pelleted by centrifugation at 300*g* for 5 min. Cells were resuspended in fresh N5 growth medium and plated in one well of a 12-well tissue culture plate per mouse. Cells from mice (3–5 animals) of same genotype from each litter (both sexes included) were pooled together. Cells were grown in N5 growth medium: DMEM/F-12 with GlutaMAX supplemented with 5% fetal bovine serum, N2 supplement (1:100), 35 mg/mL bovine pituitary extract, antibiotic-antimycotic (1:100), 20 ng/mL epidermal growth factor (EGF), and 20 ng/mL basic fibroblast growth factor (bFGF). For routine passaging, cells were grown to hyper confluence and then dissociated using 0.25% trypsin-EDTA for 3–5 min in the incubator. This was diluted with an equal volume of growth medium, and the cells were dissociated by trituration. Cells were pelleted as described above, then resuspended in fresh culture medium to plate in 2 times the original culture area.

For differentiation, at passage 4–5 cultures were grown to hyperconfluence and then the medium was replaced with the differentiation media (N5 media without EGF or FGF and 2% serum) for 5 days.

### METHOD DETAILS

#### *In vitro* transcription and purification

The *in vitro* transcription and semi-native purification of *Pnky* RNA was performed as previously described in.^27^ In brief, RNAs were transcribed by runoff transcription using T7 RNA polymerase in a buffer containing 12mM MgCl_2_, 40mM Tris-Cl pH8, 2mM Spermidine, 10mM NaCl, 0.01% Triton X-100, 10mM DTT, 5μL SUPERase-In and 3.6mM of each NTP. The reactions were incubated at 37°C for 2 h. Thereafter, 2U of TURBO DNase (2U/ul, Life Tech) was added and the mixture was incubated at 37°C for 30 min. To chelate excess divalent ions, 5μL of 0.1M EDTA was added. The RNA was buffer exchanged into filtration buffer (50mM MOPS-KOH pH 7, 150mM KCl) using a 100-kDa Amicon Ultra filtration column at room temperature. The RNA was purified using a self-packed 24mL Sephacryl S-400 gel filtration column equilibrated in filtration buffer. RNA from the peak fraction was folded by incubating with 15mM MgCl_2_ at 37°C for 40 min.

#### Sedimentation velocity- analytical ultracentrifugation

SV-AUC experiments were performed with a Beckman XL-1 centrifuge and an AN-50 Ti Analytical Rotor. For each condition, 500μL of the RNA at an A260 of 0.6 was used. RNA was folded as described above. For each condition, a 500μL of buffer was also prepared. AUC cells were cleaned thoroughly with RNase Zap and 100% ETOH, dried, and assembled according to the manufacturer’s instructions. Using a round gel loading tip, 2 × 210μL of buffer was added to the left side of the cell chamber. Similarly, 2 × 195μL of RNA was added to the right side of the cell chamber. Cells were sealed according to the manufacturer’s instructions, placed into the rotor, and equilibrated to 20°C for 1.5–2 h under vacuum. Thereafter, cells were spun at 25,000 rpm for 16 h for a total of 120 scans. Data was analyzed in Sedfit^73^ using the continuous distribution model Hydrodynamic radii (R_h_) were calculated in Sedfit using a partial specific volume of 0.53 cm^3^/g and a hydration of 0.59 g/g. Buffer viscosity and density were estimated using Sednterp and figures were made using Gussi c(s).

#### *In vitro* SHAPE

For probing, 2μg of RNA was incubated with 15mM MgCl_2_ in a final volume of 20μL and incubated at 37°C for 40 min. Thereafter, 18μL of folded RNA was transferred to a tube containing either 2μL of 100mM 1M7 (final concentration 10mM) or pure DMSO as a control and incubated at 37°C for 10 min followed by quenching with 2μL of 1M DTT. All RNA samples were purified using a Zymo RNA clean and concentrator kit according to manufacturer’s protocol and eluted in 15μL of nuclease-free water.

#### In-cell SHAPE

SVZ NSCs (*Pnky*^+/+^; BAC-*Pnky*) were grown in N5 growth medium were used. Approximately 70–90 million cells were used for each probing condition. For all probing conditions, media was aspirated, cells were washed once with DPBS,and dislodged using Accutase. The cells were collected and centrifuged at 300 g × 5 min at 25°C. The supernatant was removed and to every ~10 million cells 900μL of DPBS was added. Cells were resuspended by pipetting up and down to ensure a homogenous cell suspension. 100 μl of freshly dissolved 1M7 (in DMSO) to a final concentration of 50mM or an equivalent volume of only DMSO (as control) was added to the cells. The reaction mixture was incubated at 37°C for 5 min and pelted at 300 g × 5 min at 25°C.

The resulting cell pellet was resuspended in nuclear fractionation buffer (10mM Tris-HCl, 140mM NaCl,1.5mM MgCl_2_, 0.5% (w/v) Igepal and 10mM Ribonucleoside vanadyl complex) and incubated on ice for 5–10 min. The mixture was centrifuged 1000*g* × 5min at 4°C. The supernatant (cytoplasmic fraction) was discarded, and the procedure was repeated two more times. In the final addition of the fractionation buffer, 0.5% (w/v) sodium-deoxycholate was added. The nuclear pellet was resuspended in Trizol and extracted using Direct-zol RNA Purification Kits (Zymo Research) according to manufacturer’s protocol. The RNA was eluted in ME buffer (8mM MOPS pH 6.5, 1mM EDTA pH 8.5). Nuclear RNA was ribosome depleted using ThermoFisher (K155001) Ribominus kit according to manufacturer’s protocol.

#### MaP reverse transcription

For each probing condition, ~2000ng of IVT RNA or ~800–1000ng of nuclear enriched, ribodepleted RNA was mixed with 2 pmol of RT primer in a final volume of 10μL and annealed at 90°C for 1 min followed by 30°C for 2 min. Thereafter, 20U/μl of Superscript II, 2U/μl of SUPERase-In RNase inhibitor, and SHAPE-Map Buffer (50mM 1M Tris-HCl pH 7.5, 75mM KCl, 10mM DTT, 0.5mM dNTPs, 6mM Mn^2+^) were added and the RT reactions were incubated at 42°C for 3 h. The resulting cDNA was purified using a 1.8:1 ratio of AMPure XP beads to sample according to manufacturer’s protocol. Purified cDNA was eluted in 12μL of nuclease-free water.

#### PCR and amplicon generation

Next, amplicons were generated by using 0.5mM of gene-specific forward and reverse PCR primers, 1x Q5 reaction buffer, 0.2mM dNTPs, and 0.02U/μl Q5 HF Hot start DNA Polymerase and 10μL of purified cDNA in a final volume of 50μL. Touch down PCR cycles were used to enhance specificity with a final annealing temperature of 63°C for Amplicon 1 or 60°C for Amplicon 2. PCR samples were purified using 1:1 AMPure XP beads to sample ratio and eluted in 12μL of nuclease-free water.

#### SHAPE sequencing library prep

Amplicon concentrations were determined using a Qubit dsDNA kit according to manufacturer protocol and diluted to 0.2ng/ul. To generate sequencing libraries the NexteraXT DNA library prep kit was used with 1/5 of the manufacturer’s recommended volumes. Libraries were once more cleaned using a 1:1 AMPure beads to sample ratio.

#### Sequencing

Library concentrations were determined using a Qubit dsDNA HS Assay Kit and a BioAnalyzer High Sensitivity DNA Analysis. Libraries were diluted, pooled and sequenced using a NextSeq 500/550 or NextSeq 2000 platform.

#### SHAPE-MaP data analysis

The sequencing data was analyzed using ShapeMapper2,^70^ aligning reads to mouse *Pnky* transcript. SHAPE reactivities were computed by the ShapeMapper2 script by calculating mutation rate differences between SHAPE-modified and unmodified samples for each nucleotide of mouse *Pnky* and then normalizing the values into a final ~0–4 reactivity scale.

#### Secondary structure prediction

ShapeKnots^49^ was first used in 600-nt windows to scan *Pnky* transcript for the presence of pseudoknots using *in vitro* derived SHAPE reactivities, in addition to a prediction using the full-length context. A pseudoknot was judged plausible if both strands of pseudoknotted helix had low (<0.4) SHAPE reactivities. A plausible pseudoknot was then used as constraints for the final secondary structure computation. A consensus secondary structure of the full-length mouse lncRNA *Pnky* was obtained using SuperFold 1.0^24^ with default settings along with *in vitro* SHAPE reactivities and the identified pseudoknot constraint.

#### Identification of low SHAPE-low Shannon Entropy regions

To identify regions in the *Pnky* transcript that were both highly structured and stably folded, we used in-cell SHAPE reactivity and Shannon entropy data across two replicates. SHAPE reactivity profiles were generated using ShapeMapper2.0, as described above. Shannon entropy data was obtained from base-pairing probabilities that are calculated from SuperFold. We then averaged the two replicates of SHAPE reactivity and Shannon entropy profiles to generate a consensus SHAPE and Shannon entropy dataset. Local median SHAPE reactivity and Shannon entropy was calculated using a 28-nt sliding window. We also calculated the global median SHAPE reactivity and Shannon entropy, which was subtracted from each local median. The resulting data analysis was subsequently used to identify regions where both the local SHAPE and Shannon entropy were below the global median.

#### deltaSHAPE

To compare changes between the IVT and in-cell SHAPE-MaP derived *Pnky* structures, we used deltaSHAPE.^60^ We first averaged the two in-cell and two *in vitro* SHAPE MaP replicates to create one consensus SHAPE-MaP profile for each condition respectively. We then calculated the difference between the in-cell and *in vitro* average SHAPE reactivities (in-cell – *in vitro*) using default parameters in deltaSHAPE. The output identified statistically significant changes in SHAPE reactivity between the *in cellulo* and IVT datasets.

#### Terbium probing

Terbium probing was performed by incubating 18μL of the *in vitro* transcribed and folded RNA with 2μL of 5mM TbCl_3_ stocks prepared in monovalent buffers (50mM MOPS-KOH pH 7, 150mM KCl) to a final concentration 0.5mM or 2μL of monovalent buffer (negative control) for 10 min at 25°C. All reactions were quenched with the addition of 3μL of 50mM EDTA pH 8 and cleaned using a Zymo RNA clean and concentrator.

#### Terbium library preparation

For each probing condition, the RNA was mixed with 0.5pmol of gene-specific primers and brought to a volume of 7.5μL. To anneal primers, the mixture was heated at 90°C for 1 min followed by 30°C for 2 min. To initiate reverse transcription, 0.5 μl of Marathon RT,^74^ 10μL of 2x Marathon Ammonium Sulfate buffer (100 mM Tris-HCl pH 8.3, 400 mM (NH_4_)_2_SO_4_, 4 mM MgCl_2_, 10 mM DTT and 40% glycerol), 1μL of 10mM dNTP mix (NEB), and 1 μl of 10mM DTT were added and incubated at 42°C for 15 min. RNA was degraded with the addition of 1μL of 3M KOH, heated to 95°C for 5 min and snap cooled to 4°C for 5 min. Thereafter, 1μL of 3M HCl was added to neutralize the reaction. cDNA products from RT were purified using AMPure XP beads by adding 1.2x bead to sample ratio and incubating at room temp for 10 min. The beads were captured using a magnetic rack for 5 min and washed 3 times with 180μL of fresh 80% ETOH. The beads were air dried for 5 min and resuspended in 12μL of water to elute the cDNA. Thereafter, 3′ adaptor ligation was performed by mixing 8μL of purified cDNA with 0.2μL of 50μM 3′ adaptor (TruSeq cDNA Ligation adaptor) 1μL of 10mM ATP, 2μL of T4 RNA Ligase buffer, 8μL of 50% PEG 8000. The mixture was incubated at 25°C for 16 h, followed by enzyme deactivation at 65°C for 15 min. Ligated products were purified with AMPure XP beads using a 1.2x bead to sample ratio. The products were PCR amplified 9 cycles with Q5 HF DNA polymerase using Illumina TruSeq forward primer and indexed reverse primers, with cycle times of 98°C for 10 s, 62°C for 45 s, and 72°C for 60 s. PCR products were purified with 1.2x volume of AMPure XP beads.

#### Terbium data analysis

All FASTQ files were processed using Cutadapt to remove Illumina adaptor sequences and aligned to the respective RNA sequence using HISAT2 (v2.10). Stop information was extracted using the RTEventsCounter.py script.^26,75^ The probability of stop per nucleotide was calculated as the number of stops divided by the sum of the total number of readthrough events plus the number of stops. Probabilities were background subtracted against a no probe control. The data was normalized and scaled as in.^26^

#### LNA and gapmer ASO design and synthesis

LNAs were designed with three consecutive LNA bases at the 5′ and 3′ ends with stretches of up to three consecutive unlocked bases. The design was based on thermodynamic properties such as length, % GC content and LNA:RNA duplex T_m_ on the Qiagen LNA T_m_ calculator. All LNA oligos were synthesized on a MerMade 12 RNA-DNA synthesizer (BioAutomation) using DNA and LNA phosphoramidites (TxBio) and Glen UnySupport 1000 (GlenResearch). The synthesis cycle was modified to increase oxidation step to 3 min as recommended by the manufacturer. Removal of oligonucleotides from the polymer support and base deprotection were carried out in 28–30% aqueous ammonium hydroxide: 40% aqueous methylamine (1:1) at 65°C for 2 h. Then, the resulting oligo solutions were dried using Speedvac, redissolved in 750 μL of sterile deionized water, and desalted using GlenPak 1.0 columns (Glen Research). Scrambled LNA was designed for control. Gapmer ASOs (negative control and against *Pnky* RNA) were designed using the Qiagen Antisense LNA Gapmer design tool and procured from Qiagen.

#### LNA and gapmer ASO transfection

SVZ NSCs (at passage 4–5) were plated in poly-D-lysine and laminin coated 24-well culture plates (20–25,000 cells per well) for RNA extraction or 96 well Mattek (P96GC-1.5–5-F, No. 1.5 Coverslip | 5 mm Glass Diameter) glass bottom plate (10–12,000 cells per well) for differentiation and imaging in N5 proliferation medium, one day before transfection. Transfection was performed in 250μL total volume for 24-well plates and 100μL total volume for 96 well plates such that the final concentration of the LNA ASO is 100nM and Gapmer ASO is 50nM. Appropriate volume of LNA or Gapmer ASO was diluted in Opti-MEM reduced serum media from 20μM stock. Oligofectamine reagent (Invitrogen Cat #12252011) was diluted in the Opti-MEM media and incubated for 5–10 min. The diluted oligonucleotides were combined with diluted Oligofectamine, mixed and incubated for 15–20 min at room temperature, as per manufacturer’s protocol. While complexes are forming, the growth media is removed and replaced with 200 μl and 80 μl of N5 media without serum respectively. The transfection reagent with the LNA or Gapmer ASOs are added to the cells and incubated for 4 h at 37°C in the CO_2_ incubator. Thereafter, N5 media with 3X the normal concentration of the serum is added without removing the transfection mixture.

#### NSC differentiation assay

21–24 h post transfection with LNA or ASO, the N5 media with the oligonucleotides is removed and the differentiation media N6 (without the growth factors) is added to each well. Cells are allowed to undergo differentiation for 5 days.

#### Immunocytochemistry (ICC)

After 5 days of differentiation, the cells are washed 1X with PBS and fixed with 4% paraformaldehyde for 30 min at RT. Post fixation, cells are washed 3X with PBS. Citrate antigen retrieval was performed using heated 10 mM sodium citrate (pH 6.0) for 10–15 min in a slide box. The sodium citrate was removed, and the slides were rinsed in PBS. ICC were performed using blocking buffer consisting of PBS with 1% BSA, 0.3-M glycine, 0.3% Triton X-100 and 10% normal goat serum. Cells were incubated in blocking buffer for 1 h at RT, followed by the incubation in primary antibody Tuj1 diluted (1:1000) in blocking buffer for 2 h at RT. Slides are washed 3X in PBS at RT for 5 min, followed by the incubation in secondary antibodies (Alexa Fluor antibodies, 1:500) and DAPI (1:500) diluted in blocking buffer for 45 min at RT. This is followed by 3X wash in PBS and then coverslipped using the AquaMount.

#### Microscopy imaging and quantification

Cells were imaged using Leica Epifluorescence microscope. For each transfection condition, experiments were performed in independent triplicates. For each well, 3–4 non-overlapping fields of view were imaged. All images were processed using Fiji and quantified using the Imaris software (spots function where the diameter of the spot was set to 8.00) by an experimenter blinded to the target location of LNA probes on *Pnky* RNA. Total number of DAPI and Tuj1 positive spots were counted to obtain % Tuj1+ normalized to total DAPI positive nuclei.

#### RNA isolation

21–24 h post transfection with LNA or ASO, total RNA was isolated from cultured cells using Trizol (Thermo Fisher Scientific) and purified using Direct-zol RNA Purification Kits (Zymo Research). DNase digestion was performed as suggested.

#### RT-qPCR

cDNA synthesis was performed using the Transcriptor first Strand cDNA synthesis kit. qRT-PCR was performed using SYBR Green on a Roche LightCycler 480 II. Relative gene expression was calculated using the ^ΔΔ^Ct method, using GAPDH as a housekeeping gene for differential gene expression analyses. All RT-qPCR assays were performed using technical triplicate wells. *Pnky* RT-qPCR primers span the exon 1 and 2 junction-region that is also targeted by LNAs 11–19. Binding of LNA ASOs have been reported to inhibit reverse transcription.^76,77^ Therefore, to for the RT-qPCR readout for LNAs 11–19 transfectants, we designed another primer pair (spanning exon 2 and 3) to investigate *Pnky* transcript levels in these cells.

#### Figure preparation

Graphs were generated using PRISM (GraphPad). Figures were generated using Adobe Illustrator and schematics using BioRender.

#### Quantification and statistical analysis

The statistical information of each experiment, including the statistical methods, the *p* value and sample numbers (n) are shown in figure legends. All experiments were performed at least two times, with similar results. Statistical significance was expressed as a *p* value.

## Supplementary Material

1

SUPPLEMENTAL INFORMATION

Supplemental information can be found online at https://doi.org/10.1016/j.celrep.2026.117451.

## Figures and Tables

**KEY RESOURCES TABLE:** 

REAGENT or RESOURCE	SOURCE	IDENTIFIER

Antibodies		

Anti Tubulin β3 (Tuj1)	Biolegend	Cat# 801201; RRID: AB_2313773
Goat anti-Mouse IgG2a Cross- Adsorbed Secondary Antibody, Alexa Fluor^™^ 488	ThermoFisher	Cat# A-21131; RRID: AB2535771

Chemicals, peptides, and recombinant proteins		

1-methyl-7-nitroisatoic anhydride (1M7)	New England Discovery Partners	N/A
TbCl_3_	Sigma-Aldrich,	Cat# 212903
MgCl_2_	Sigma-Aldrich	Cat# S255777
NaCl	Sigma-Aldrich	Cat# S9888
KCl	Sigma-Aldrich	Cat# 409316
T7 RNA Polymerase	Purified in-house	N/A
SUPERase-In	Thermo-Fisher	Cat# AM2694
Spermidine	Sigma-Aldrich	Cat# 85558
EDTA	Thermo-Fisher,	Cat# AM9260G
Tris buffer	Thermo-Fisher,	Cat# 15504020
MOPS	Sigma-Aldrich,	Cat# M1254
HEPES	Sigma-Aldrich,	Cat# H7006
MES	Sigma-Aldrich,	Cat# M0895
TURBO DNase	Thermo-Fisher,	Cat# AM2238
Complete Protease Inhibitor Cocktail EDTA-free	Roche,	Cat# 05056489001
DMSO	Sigma	Cat# D2650
DTT	Sigma	Cat# 43816-10ML
Igepal	Millipore Sigma	Cat# I8896-50ML
Ribonucleoside vanadyl complex	NEB	Cat# S1402S
SuperScript II Reverse Transcriptase	Thermo Fisher Scientific	Cat# 18064014
Q5 HF Hot start DNA Polymerase	NEB	Cat# M0491
dNTPs	NEB	Cat# N0447
Marathon RT	Made in-house	N/A
T4 RNA Ligase buffer	NEB	Cat# M02024
Q5 reaction buffer	NEB	Cat# B9027
LightCycler^®^ 480 SYBR Green I Master 5X	Roche	Cat# 04707516001
Laminin	Millipore-Sigma	Cat# L2020-1MG
L15 media	ThermoFisher	Cat# 11415064
DMEM/F-12 GlutaMAX	ThermoFisher	Cat# 10565018
0.25% Trypsin-EDTA	ThermoFisher	Cat# 25200056
Fetal Bovine Serum - TET Tested, Heat Inactivated	R&D Systems	Cat# S10350H
N2 Supplement	ThermoFisher	Cat# 17502048
Bovine Pituitary Extract	ThermoFisher	Cat# 13028014
Antibiotic-Antimycotic	ThermoFisher	Cat# 15240062
Epidermal Growth factor (EGF)	PeproTech	Cat# AF-100-15
Basic Fibroblast growth Factor (b-FGF)	PeproTech	Cat# 100-18 B
Opti-MEM reduced serum media	ThermoFisher	Cat# 31985062
Oligofectamine^™^ Transfection Reagent	Invitrogen	Cat# 12252011
AMPure Beads	Beckman Coulter	Cat# A63880
TRIzol^™^ Reagent	Thermo Fisher Scientific	Cat# 15596018
Chloroform: Isoamyl alcohol 24:1	Millipore-Sigma	Cat# C0459-1PT
HaltTM Protease Inhibitor	Thermo Fisher Scientific	Cat# 78438
Normal goat serum	Jackson Immunoresearch Laboratories	Cat# 005-000-121

Critical commercial assays		

RNA clean and concentrator kit	Genesee	Cat# R1013
Direct-zol RNA purification kit	Zymo Research	Cat# R2052/62
RiboMinus^™^ Human/Mouse Transcriptome Isolation Kit	ThermoFisher	Cat# K155001
Transcriptor First Strand cDNA synthesis Kit	Roche	Cat# 04897030001
Qubit dsDNA HS Assay Kit	Thermo-Fisher,	Cat# Q32851
BioAnalyzer High Sensitivity DNA Kit	Agilent	Cat# 5067-4626
NextSeq 500/550 Mid Output kit v2.5 (150 cycles)	Illumina	Cat# 20024904
NextSeq 1000/2000 P1 Reagents (300 Cycles)	Illumina	Cat# 20050264

Deposited data		

*In vitro* SHAPE MaP *Pnky*	This study	BioProject ID: PRJNA1263515
In-cell SHAPE MaP *Pnky*	This study	BioProject ID: PRJNA1263515
Tb-seq *Pnky (in vitro* transcribed*)*	This study	BioProject ID: PRJNA1263515

Experimental models: Organisms/strains		

Mouse: wild type: C57BL/6J	The Jackson Laboratory	–
Mouse: *Pnky*^null^	(Andersen et al. 2019)^7^	–
Mouse: BAC^*Pnky*^	(Andersen et al. 2019)^7^	–

Oligonucleotides		

LNA ASOs, See Table S3	This study	N/A
Antisense LNA GapmeR Control - Negative Control A	Qiagen	Cat# 339515 LG00000002-DDA
Antisense LNA GapmeR Standard-MMPNKY_6	Qiagen	Cat# 339511 LG00832266-DDA
*Pnky* MaP RT primers See Table S5	This study	N/A
RT-qPCR primers and genotyping primers, see Table S5	This study	N/A

Recombinant DNA		

pGEM-T mouse *Pnky*	This study	N/A

Software and algorithms		

SedFit	https://sedfitsedphat.github.io/	https://sedfitsedphat.github.io/
Sednterp and Gussi	(Philo et al.)^69^	–
ShapeMapper2	(Busan et al.)^70^	https://github.com/Weeks-UNC/shapemapper2
ShapeKnots	(Hajdin et al.)^49^	http://github.com/catacs/ShapeKnots-GUI
Delta SHAPE	(Smola et al.)^60^	https://github.com/Weeks-UNC/deltaSHAPE
SuperFold	(Smola et al.)^24^	https://github.com/Weeks-UNC/Superfold
Cutadapt	(Marcel et al.)^71^	https://github.com/marcelm/cutadapt
HISAT2	–	https://daehwankimlab.github.io/hisat2/
RTEventsCounter.py script	(Patel et al.)^26^	https://github.com/pylelab/Tb-seq.
Fiji	(Schidelin et al.)^72^	https://github.com/fiji/fiji
Imaris10	Oxford Instruments	https://imaris.oxinst.com/versions/10
Prism 10	Graphpad Software	N/A
Adobe Illustrator	Adobe Systems Inc.	N/A
Biorender	Biorender	N/A

Other		

Glass bottom Poly-*d*-lysine coated 96-well plates No. 1.5	MatTek Corporation	Cat# P96GC-1.5-5-F
100-kDa Amicon Ultra Filtration column	Sigma Aldrich	Cat # UFC5100
24 mL Sephacryl S-400 gel filtration column	Hand packed using Cytiva 10/300 GL column	N/A
GlenPak 1.0 Columns	Glen Research	Cat# 61-5010

## Highlights

- *Pnky* folds into highly structured conformation
- *Pnky* is a compact lncRNA with evidence of tertiary interactions
- *Pnky* structure in neural stem cells is similar to its *in vitro* folded conformation
- LNA ASO perturbations identify structured regions of *Pnky* involved in neuronal differentiation
