# Supplementary material for: Pnky lncRNA secondary structure maps identify functional regions that control neurogenesis in neural stem cells
Source: Cell Rep. Author manuscript; Available in PMC 2026 Aug 6. (PMC13446462; doi:10.1016/j.celrep.2026.117451)
Supplement: Supplementary file 1 [file NIHMS2190726-supplement-1.pdf]

Cell Reports, Volume *45*

Supplemental information

Pnky lncRNA secondary structure maps identify functional regions that control neurogenesis in neural stem cells

Parna Saha, Shivali Patel, Lucille H. Tsao, Rafael CA. Tavares, Hyeonseok Choi, Rebecca E. Andersen, Anna Marie Pyle, and Daniel A. Lim

A

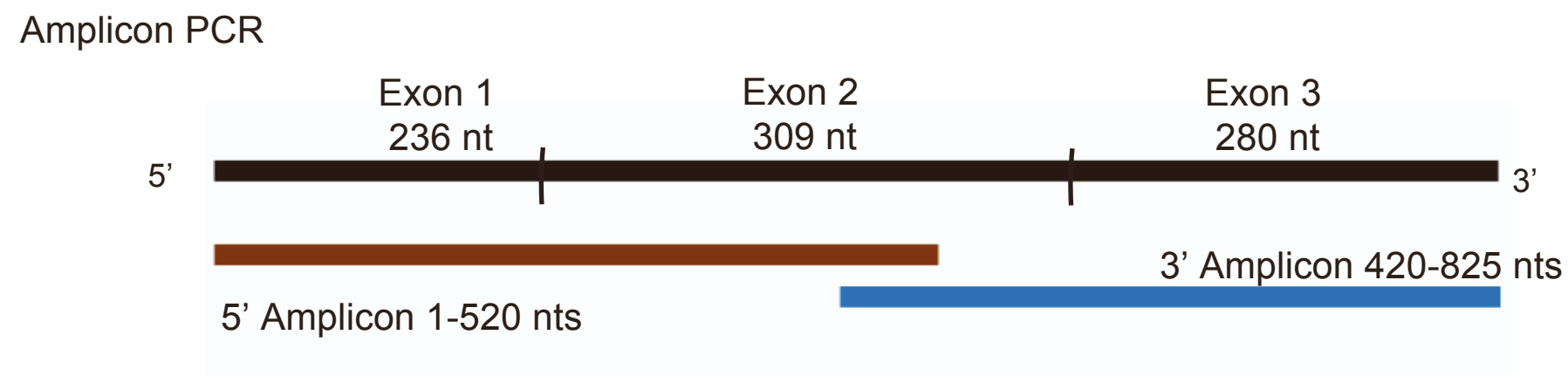

B

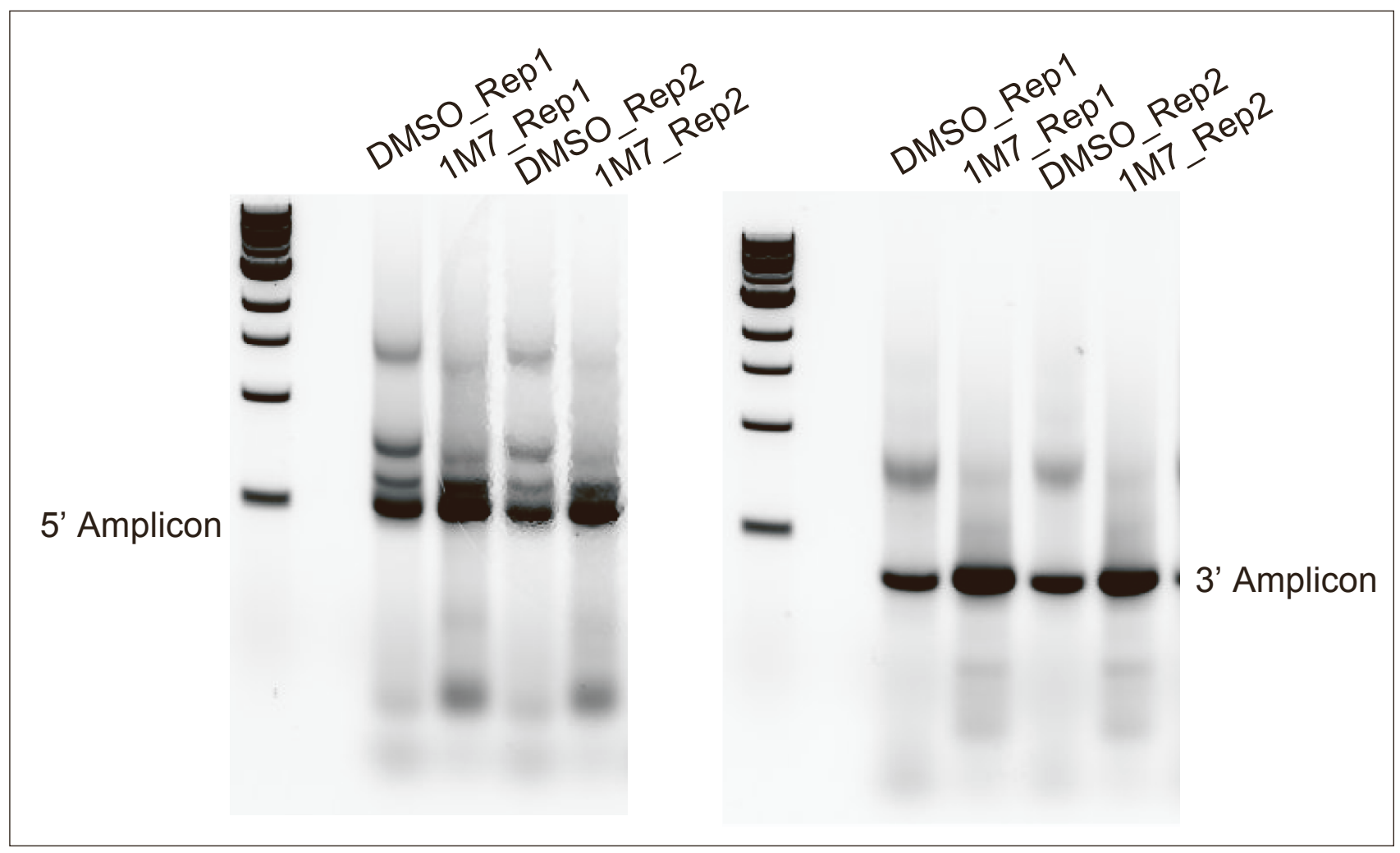

Figure S1:Amplicons for the *in vitro* SHAPE MaP related to Figure.2

(A) Schematic for amplicon PCR primer design – two sets of overlapping primers were designed to amplify the fully spliced *Pnky* RNA (825bp).

(B) Gel image showing the products of the amplicon PCR for the two replicates of DMSO and 1M7 treated samples.

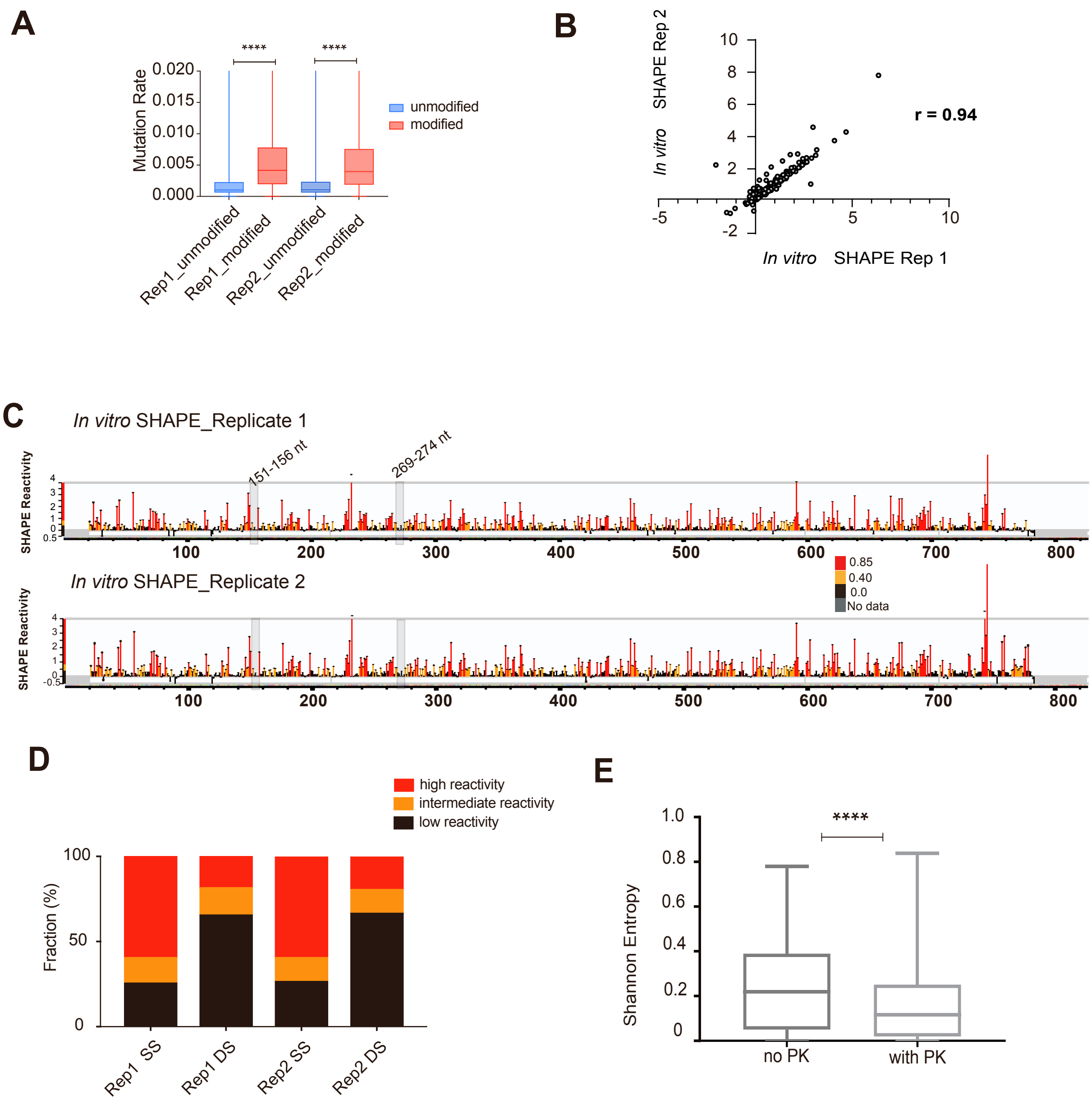

Figure S2. Quality metrics for the *in vitro* SHAPE MaP datasets, related to Figure. 2
(A) Comparison of mutation rates of 1M7 modified and unmodified samples for n= 2 independent replicates. Mean +/- SD, ****p <0.0001; unpaired two-tailed Student's t test.
(B) Correlation plot of normalized SHAPE reactivities from 2 replicates. Linear regression fit to the data is represented by the line. Pearson's correlation for the dataset is indicated.
(C) SHAPE reactivity profile for 1M7 treated replicates of *in vitro* transcribed (IVT) *Pnky* RNA. Nucleotides involved in the pseudoknot interaction (151-156 and 269-274) are highlighted in grey.
(D) Strandedness of low, moderate and highly SHAPE reactive nucleotides in the two replicates of in vitro SHAPE datasets.
(E) Shannon entropy is significantly lower in the model with the pseudoknot included.
***p <0.001; unpaired two-tailed Student's t test. See also Table S1.

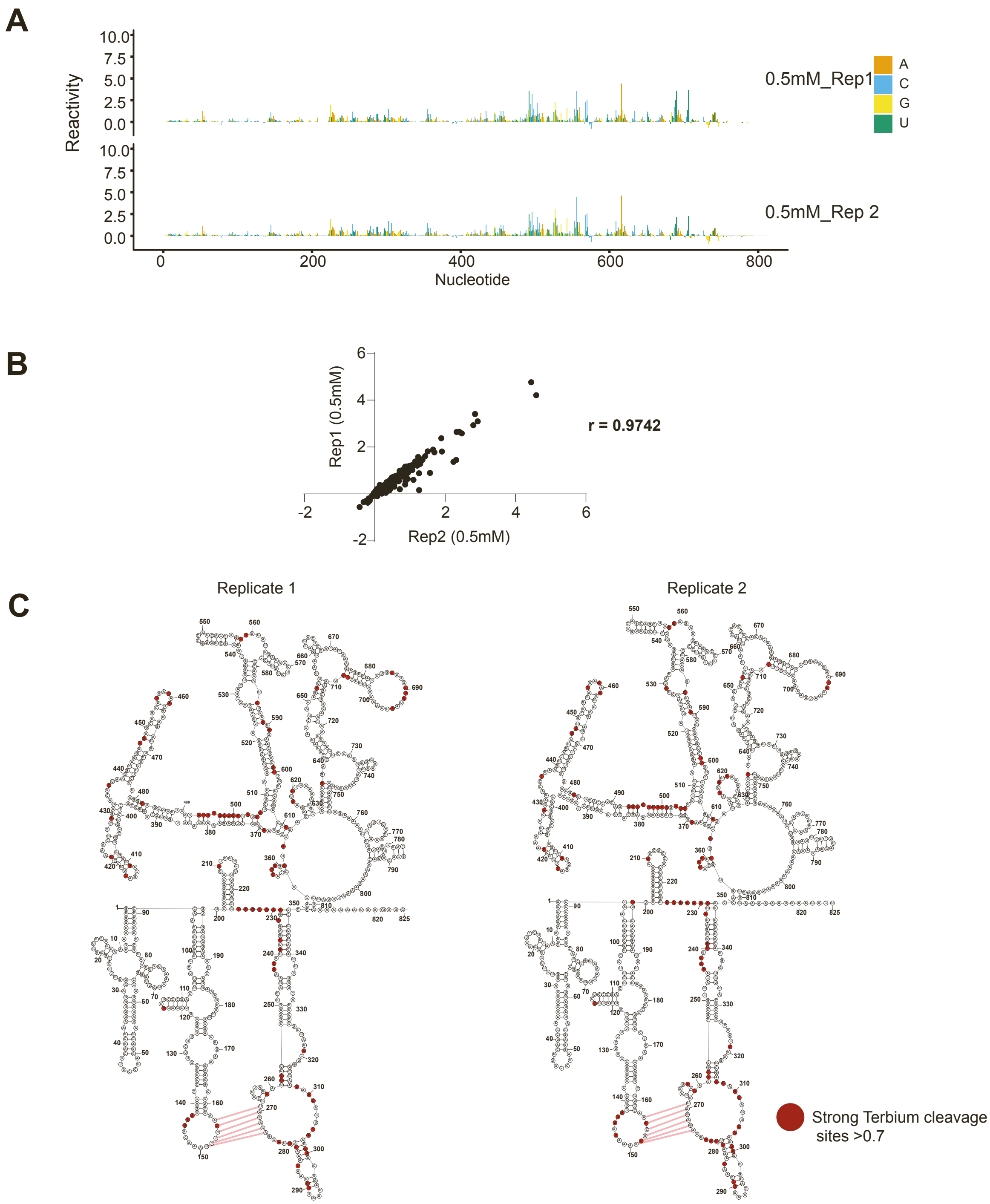

Figure S3.Quality metrics for the terbium sequencing datasets, related to Figure 3.
(A) Bar plot showing reactivity profile obtained when probing in vitro transcribed and folded *Pnky* RNA for the two independent replicates.
(B) Correlation plot of normalized terbium reactivities from 2 replicates. Linear regression fit to the data is represented by the line. Pearson's correlation for the dataset is indicated.
(C) Secondary structure of the *Pnky* RNA displaying sites of strong Tb^{+3}cleavage (red) for the two independent replicates.

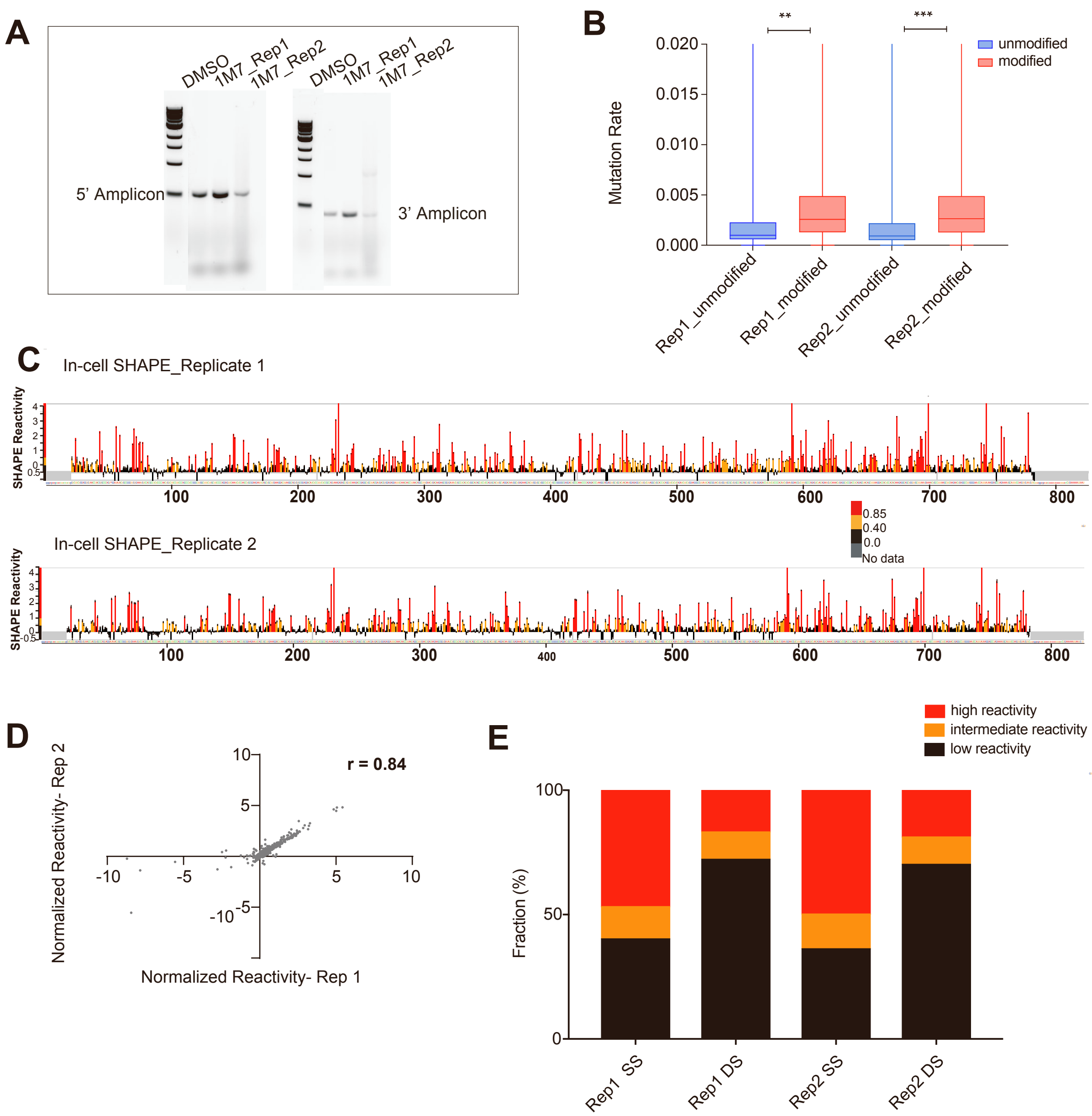

Figure S4. Quality metrics for the in-cell SHAPE datasets, related to Figure 4.

(A) Gel image showing the products of the amplicon PCR for the two independent biological replicates of DMSO and 1M7 treated samples.

(B) Comparison of mutation rates of 1M7 modified and unmodified samples for two independent replicates. Mean +/- SD, ***p* < 0.01, ****p* < 0.001; unpaired two-tailed Student's t test.

(C) showing the SHAPE reactivity profile for 1M7 treated replicates for the two independent in-cell SHAPE experiments.

(D) Correlation plot of normalized SHAPE reactivities from two biological replicates from neural stem cells. Line represents the linear regressions fit to the data. Pearson's correlation for thedatasets is shown.

(E) Stranded-ness of low, moderate and highly SHAPE reactive nucleotides in the two biological replicates of in-cell SHAPE experiments. See also Table S1.

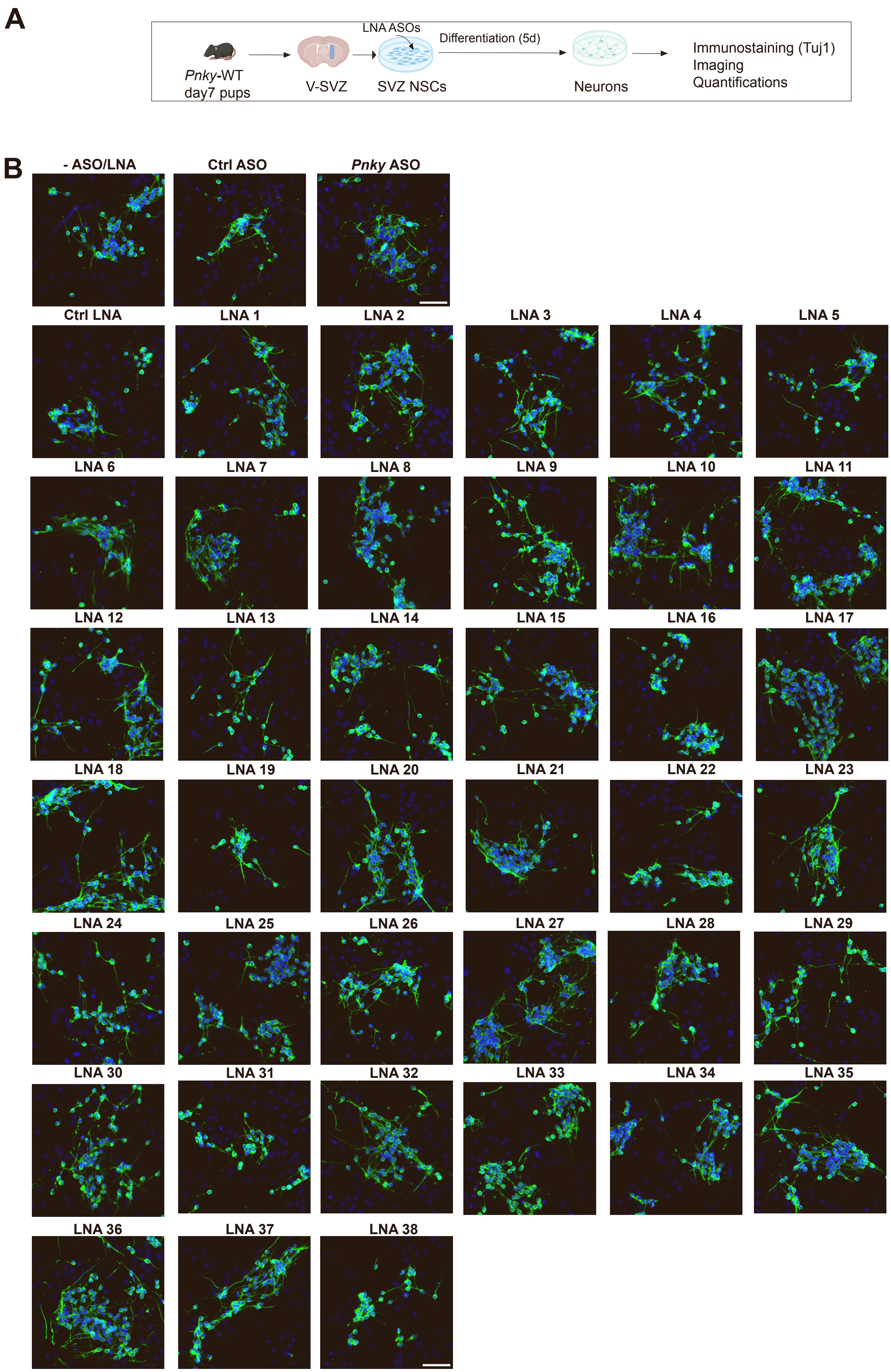
A
LNA ASOs
Differentiation (5d)
Pnky-WT
day7 pups
V-SVZ
SVZ NSCs
Neurons
Immunostaining (Tuj1)
Imaging
Quantifications
B
- ASO/LNA
Ctrl ASO
Pnky ASO
Ctrl LNA
LNA 1
LNA 2
LNA 3
LNA 4
LNA 5
LNA 6
LNA 7
LNA 8
LNA 9
LNA 10
LNA 11
LNA 12
LNA 13
LNA 14
LNA 15
LNA 16
LNA 17
LNA 18
LNA 19
LNA 20
LNA 21
LNA 22
LNA 23
LNA 24
LNA 25
LNA 26
LNA 27
LNA 28
LNA 29
LNA 30
LNA 31
LNA 32
LNA 33
LNA 34
LNA 35
LNA 36
LNA 37
LNA 38

Figure S5: LNA and gapmer ASO transfection in the *Pnky*-WT NSCs and differentiation assay. Related to Figure 5.

(A) Schematic of the LNA and Gapmer ASO transfection in the NSCs and differentiation assay.

(B) Representative images of the Tuj1 ICC with DAPI nuclear stain in 5 days (5d) differentiated *Pnky*-WT V-SVZ neural stem cell primary culture transfected with LNA and Gapmer ASOs.

Scale bar 50um.

See also Table S3.

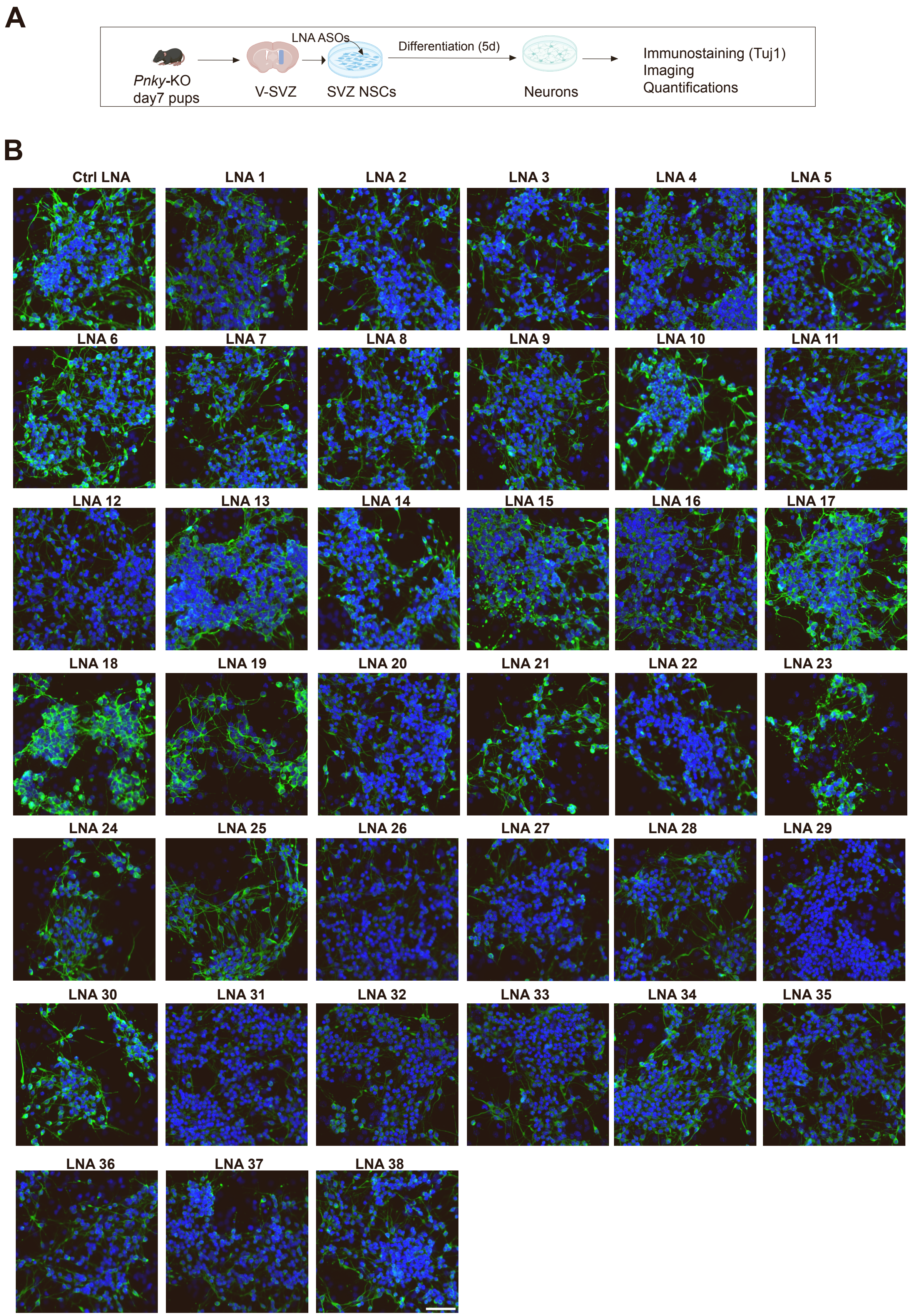
A
LNA ASOs
Differentiation (5d)
Pnky-KO
day7 pups
V-SVZ
SVZ NSCs
Neurons
Immunostaining (Tuj1)
Imaging
Quantifications
B
Ctrl LNA
LNA 1
LNA 2
LNA 3
LNA 4
LNA 5
LNA 6
LNA 7
LNA 8
LNA 9
LNA 10
LNA 11
LNA 12
LNA 13
LNA 14
LNA 15
LNA 16
LNA 17
LNA 18
LNA 19
LNA 20
LNA 21
LNA 22
LNA 23
LNA 24
LNA 25
LNA 26
LNA 27
LNA 28
LNA 29
LNA 30
LNA 31
LNA 32
LNA 33
LNA 34
LNA 35
LNA 36
LNA 37
LNA 38

Figure S6. LNA ASO transfection in the *Pnky*-KO NSCs and differentiation assay. Related to Figure 5.

(A) Schematic of the LNA ASO transfection in the NSCs and differentiation assay.

(B) Representative images of the Tuj1 ICC with DAPI nuclear stain in 5 days (5d) differentiated *Pnky*-KO V-SVZ neural stem cell primary culture transfected with LNA ASOs. Scale bar 50um.

See also Table S3.

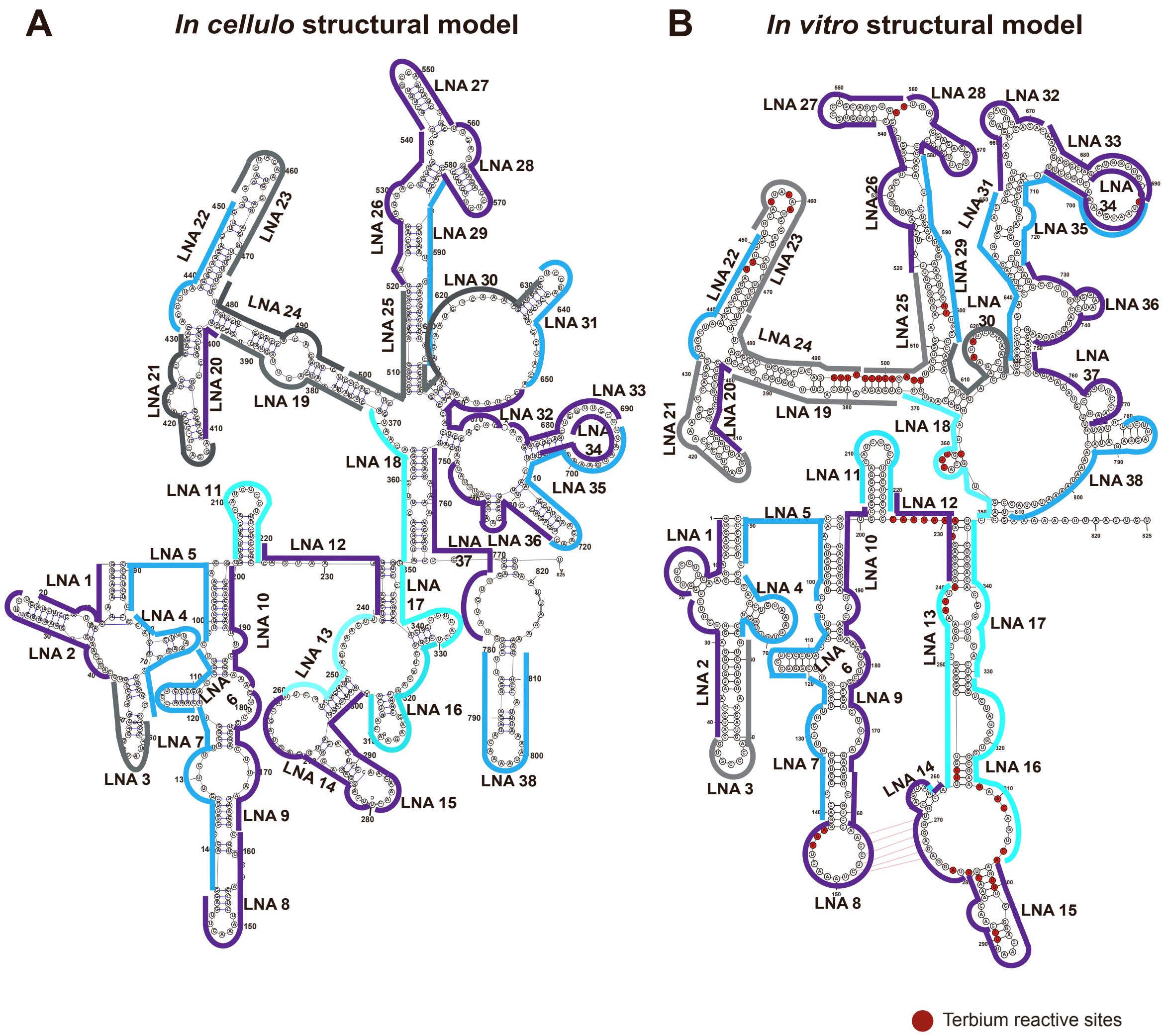

Figure S7 LNA ASO phenotypic data maps to specific structured regions of *Pnky*

(A) maps the locations of the LNA ASOs on the in-cell structural model of *Pnky* RNA.

(B) maps the locations of the LNA ASOs on the in vitro structural model of *Pnky* RNA.

'Neurogenic' LNAs in purple, 'non-neurogenic' LNAs in light blue, 'Pnky destabilizing' LNAs in teal and 'toxic' LNAs in grey.

See also Table S3.

Table S1. Quality metrics of chemical probing sequencing data (SHAPE seq), related to Figures 2 and 4.

**Libraries**	**Read depth check (%)**	**Mutation rate check (%)**	**High background check %**	**No. of highly reactive nucleotides check (%)**
**SHAPE Mapper threshold**	>80%	>50%	<5%	>8%
**In-cell Rep 1**	97.8%	85.1%	0.4%	6.7%
**In-cell Rep 2**	97.8%	90.8%	0.4%	8%
***In vitro* Rep 1**	97.8%`	94.6%	0.4%	20.3%
***In vitro* Rep 2**	97.8%	93.8%	0.4%	19.1%

Table S2. Module locations/nucleotides (in-cell vs *in vitro*), related to Figures 2 and 4.

Modules	*In vitro* model	In-cell model
1	1-91	1-91
2	92-200	92-200
3	201-224	201-224
4	232-350	232-350
5	371-505	371-505
6	507-608	507-608
7	613-774	609-783

Table S3. List of LNAs used: targeting nucleotide positions, sequence, Tm. Related to Figures 5 and 6.

LNA	Nucleotide position	Sequence	length	Tm
**Ctrl LNA**		5’+G+T+GTA+ACA+CGT+CTA+TAC+GC+C+C+A 3’	22	81
**1**	1-22	5’ +A+C+CAG+AGG+AAG+TTG+CTT+C+T+C 3’	20	78
**2**	23-42	5’ +G+T+AGG+TTA+CAC+CTC+CAG+A+A+G 3’	20	76
**3**	43-62	5’ +C+A+GTA+TAT+CCT+ACT+GGG+C+A+C 3’	20	78
**4**	63-83	5’ +C+G+TGG+ACA+TTT+CAC+AA+CC+C+G+G 3’	21	86
**5**	84-103	5' +G+G+AAG+ACA+GTT+GGG+GAG+A+G+G 3'	20	87
**6**	104-119	5’ +C+C+CGG+AGG+GCT+GGG+A+A3’	16	87
**7**	120-141	5’+C+T+GGG+GAA+GCA+AGA+AAG+CC+A+A+G 3’	22	86
**8**	142-162	5’+C+A+AGC+AGC+TGA+ATT+CTT+C+C+G 3’	20	79
**9**	162-181	5’ +C+A+GGT+TGG+AGA+TTT+GAA+C+T 3’	19	77
**10**	182-202	5’+G+C+ACA+CTG+CC+TT+AAG+TCC+T+T+T 3’	21	80
**11**	203-223	5' +C+G+GAG+AAA+GGA+GAT+GTCC+T+C+C 3'	21	84
**12**	220-239	5’ +C+A+GCT+CTC+TTT+ACT+GGC+G+G+A 3’	20	83
**13**	240-259	5' +A+A+GCA+GCC+TCG+GTC+TTT+G+A+A 3'	20	78
**14**	260-279	5’ +A+A+TCC+TCT+CCA+CGT+CAT+C+G+T 3’	20	79
**15**	280-303	5' +T+C+AAA+GCC+TGT+TAA+GGT+TGT+T+G+A 3'	23	80
**16**	304-328	5' +G+C+AGA+TAT+CAC+CGC+TTC+TTG+TC+A+G+T 3'	25	81
**17**	329-349	5' +G+A+G+GAG+CTG+TGC+CAG+AGTG+A+G 3'	21	88
**18**	350-372	5' +C+C+ATT+GTC+CTA+GCA+AGT+GCA+C+T+G 3'	23	84
**19**	373-394	5' +C+C+A+GCA+CCA+AGT+GCT+TTCT+C+A+G 3'	22	84
**20**	395-411	5'+C+G+CCC+CCG+GCA+GAGA+G+G 3'	17	89
**21**	412-431	5' +T+C+TGG+GGT+TTG+GGC+AGT+C+C+T3'	20	88
**22**	432-451	5' +G+C+AAC+TCC+TGC+CTT+AGG+G+T+C 3'	20	80
**23**	452-473	5' +A+A+GAC+CTC+TCC+ATT+GTA+GT+G+C+A 3'	22	78
**24**	474-496	5' +G+A+ACC+TGA+GGT+GCA+GCC+TAA+G+A+C 3'	23	84
**25**	497-519	5' +A+C+GCC+CTT+CCA+GAA+GAT+CTG+A+G+A 3'	23	84
**26**	520-541	5' +G+C+AAC+CAC+AGT+ACC+GCT+GA+A+T+A 3'	22	82
**27**	542-559	5' +A+G+CAG+CTG+CTG+GC+AC+A+G+C 3'	18	88
**28**	560-580	5' +G+G+GGA+GAA+GAG+GTC+TCCA+T+C+A 3'	21	85
**29**	581-604	5' +G+G+CAA+AGA+CGT+TCC+ATT+CAG+AT+G+T 3'	24	81
**30**	605-631	5' +C+T+CAG+TTT+TGA+CAT+TCT+GTA+GACT+C+T+G 3'	27	80
**31**	632-651	5' +T+G+TAG+CTC+TGA+GGT+CGG+A+G+C 3'	20	85
**32**	652-672	5' +G+T+GTG+GAG+TGG+TCC+TTAA+A+G+C 3'	21	78
**33**	673-691	5' +A+G+CAA+CCA+GCT+GCC+TC+T+T+T 3'	19	83
**34**	682-706	5’+G+G+CAG+TT+TC+ATT+AAA+GCA+ACC+A+G+C 3’	24	80
**35**	692-723	5'+A+G+TTC+TTG+AAG+CTT+AAA+GGC+AGT+TTT+CAT+T +A+A 3'	32	77
**36**	724-749	5' +C+T+TAT+AAA+TGG+ATT+CCC+CAA+GGC+C+T+C 3'	26	85
**37**	750-779	5' +A+T+ACA+ACT+TGG+AAA+TGC+ATT+TTC+TAGG+C+T+C 3'	30	77

**38**	780-811	5' +C+A+GGT+AAA+TTT+TATT+TTG+TAT+CT+CCT+AAG+A+A +C 3'	32	75
**Gapmer ASOs**				
**Ctrl ASO**		A*A*C*A*C*G*G*T*C*T*A*T*A*C*G*C		
**Pnky ASO**	516-532	T*A*C*C*G*C*T*G*A*A*T*A*A*C*G*C		

Table S4. List of LNA ASOs that overlap with the Low SHAPE- Low Shannon Entropy regions, ΔSHAPE regions and the terbium reactive nucleotides. Related to Figure 7.

Low SHAPE Low Shannon- “structured regions “	LNA ASOs that overlap with these region	
	Neurogenic	Non-neurogenic
37-48	**2**	
52-74		**4**
121-156	**8**	**7**
180-182	**9, 10**	
188-191	**10**	
193-239	**10, 12**	
391-441	**20**	**22**
443-447		**22**
652-658	**32**	

+ve ΔSHAPE nucleotides	LNA ASOs that overlap with these regions	-ve ΔSHAPE nucleotides	LNA ASOs that overlap with these regions
76-78	**4**	25-29	**2**
184-186	10	44-46	3
422-424	21	84-86	5
546-547	27	444-446	**22**
590-593	29	486-488	24
616-617	30	490-491	24
619	30	549-554	27
697-700	34/35	571-573	28
750-752	37	742-746	36

Terbium Reactive nucleotides	Neurogenic LNA ASOs	Non-neurogenic LNA ASOs
143-145	**8**	
225-231, 233	**12**	
277, 279	14	
281, 289-290, 300-301	15	
447-448		**22**
558-559	27	
599-600		29
691-692	33, 34	

Table S5. List of primers- RT, Amplicon, RT- qPCR and genotyping PCR primers

**Description**	**Sequence (5'-3')**
Pnky specific RT primer for MaP RT	GCGCAACCACAGTACCGCTGAATA
Pnky specific RT primer for MaP RT	CAGGTAAATTTTATTTTGTATCTCCTAAGAAC
Gene specific RT primer with TruSeq overhang	CAGACGTGTGCTCTTCCGATCTACAATTAATTTTTTCAGGTAAATT
TruSeq cDNA ligation 3' adaptor (for Tb-seq)	5'Phos-NNNNNNAGATCGGAAGAGCGTCGTGTAG-3'Bio
Amplicon1 Forward primer	GGGAGAAGCAACTTCCTCTG
Amplicon 1 Reverse primer	GCGCAACCACAGTACCGCTGAATA
Amplicon 2 Forward primer	CCAAACCCCAGAGACCCTA
Amplicon 2 Reverse primer	CAGGTAAATTTTATTTTGTATCTCCTAAGAAC
*Pnky*_Exon1_2 qPCR Forward primer	GGACATCTCCTTTCTCCGCC
Pnky _ Exon1_2 qPCR Reverse primer	CACCAAGTGCTTTCTCAGCC
*Pnky*_Exon2_3 qPCR Forward primer	GATCTTCTGGAAGGGCGTTATT
*Pnky*_Exon2_3 qPCR Reverse primer	TGGGCAAAGACGTTCCATT
GAPDH qPCR Forward primer	GGGAAATTCAACGGCACAGT
GAPDH qPCR Reverse primer	AGATGGTGATGGGCTTCCC
Pnky Genotyping Forward primer	TAAGCTCAAACTCCGGTCCCGGGA
Pnky Genotyping Reverse1 primer	TCAGGGACAAAGAACCAAAACGAGC
Pnky Genotyping Reverse2 primer	AATGCTCCCTCTGAGCCTCAATT
BAC Genotyping Forward primer	CACCTGCTACCTGATATAGG
BAC Genotyping Reverse primer	CCTGCTACCTGATATAGG
